# Succession of the wheat seed-associated microbiome as affected by soil fertility level and introduction of *Penicillium* and *Bacillus* inoculants in the field

**DOI:** 10.1093/femsec/fiac028

**Published:** 2022-03-14

**Authors:** Inês Nunes, Veronika Hansen, Frederik Bak, Lise Bonnichsen, Jianqiang Su, Xiuli Hao, Nelly Sophie Raymond, Mette Haubjerg Nicolaisen, Lars Stoumann Jensen, Ole Nybroe

**Affiliations:** Microbiomics and Microbe Discovery Denmark, Novozymes A/S, Biologiens Vej 2, 2880 Kgs Lyngby, Denmark; Plant and Soil Section, Department of Plant and Environmental Sciences, Faculty of Science, University of Copenhagen, Thorvaldsensevej 40, 1871 Frederiksberg C, Denmark; Section for Microbial Ecology and Biotechnology, Department of Plant and Environmental Sciences, Faculty of Science, University of Copenhagen, Thorvaldsensvej 40, 1871 Frederiksberg C, Denmark; Section for Microbial Ecology and Biotechnology, Department of Plant and Environmental Sciences, Faculty of Science, University of Copenhagen, Thorvaldsensvej 40, 1871 Frederiksberg C, Denmark; Key Laboratory of Urban Environment and Health, Institute of Urban Environment, Chinese Academy of Sciences, Xiamen 361021, China; Section for Microbial Ecology and Biotechnology, Department of Plant and Environmental Sciences, Faculty of Science, University of Copenhagen, Thorvaldsensvej 40, 1871 Frederiksberg C, Denmark; State Key Laboratory of Agricultural Microbiology, Huazhong Agricultural University; Key Laboratory of Arable Land Conservation (Middle and Lower Reaches of Yangtze River), Ministry of Agriculture, Huazhong Agricultural University, Wuhan 430070, China; Plant and Soil Section, Department of Plant and Environmental Sciences, Faculty of Science, University of Copenhagen, Thorvaldsensevej 40, 1871 Frederiksberg C, Denmark; School of Agriculture and Food Sciences, The University of Queensland, St Lucia, Queensland 4072, Australia; Section for Microbial Ecology and Biotechnology, Department of Plant and Environmental Sciences, Faculty of Science, University of Copenhagen, Thorvaldsensvej 40, 1871 Frederiksberg C, Denmark; Plant and Soil Section, Department of Plant and Environmental Sciences, Faculty of Science, University of Copenhagen, Thorvaldsensevej 40, 1871 Frederiksberg C, Denmark; Section for Microbial Ecology and Biotechnology, Department of Plant and Environmental Sciences, Faculty of Science, University of Copenhagen, Thorvaldsensvej 40, 1871 Frederiksberg C, Denmark

**Keywords:** fertilizer amendment, long-term field trial, microbial inoculants, nitrogen and phosphorus cycling genes, seed microbiota, wheat seed microbiome

## Abstract

During germination, the seed releases nutrient-rich exudates into the spermosphere, thereby fostering competition between resident microorganisms. However, insight into the composition and temporal dynamics of seed-associated bacterial communities under field conditions is currently lacking. This field study determined the temporal changes from 11 to 31 days after sowing in the composition of seed-associated bacterial communities of winter wheat as affected by long-term soil fertilization history, and by introduction of the plant growth-promoting microbial inoculants *Penicillium bilaiae* and *Bacillus simplex*. The temporal dynamics were the most important factor affecting the composition of the seed-associated communities. An increase in the relative abundance of genes involved in organic nitrogen metabolism (*ureC* and *gdhA*), and in ammonium oxidation (*amoA*), suggested increased mineralization of plant-derived nitrogen compounds over time. Dynamics of the phosphorus cycling genes *ppt*, *ppx* and *cphy* indicated inorganic phosphorus and polyphosphate cycling, as well as phytate hydrolysis by the seed-associated bacteria early after germination. Later, an increase in genes for utilization of organic phosphorus sources (*phoD*, *phoX* and *phnK*) indicated phosphorus limitation. The results indicate that community temporal dynamics are partly driven by changed availability of major nutrients, and reveal no functional consequences of the added inoculants during seed germination.

## Teaser

Development of the wheat seed-associated microbiome and its functional potential of nitrogen and phosphorous cycling.

## Introduction

Substantial efforts have been devoted to the characterization of plant-associated microbiomes from the rhizosphere and phyllosphere (Mendes *et al*. 2013, Compant *et al*. 2019). In comparison, bacterial communities in or on the seed and in the surrounding spermosphere have only received limited attention even though they represent the starting point for assembly of other plant microbiomes (Torres-Cortés *et al*. 2018). Interactions between the plant and its associated microbiota are crucial during the dynamic phase of seed germination and seedling development (Nelson *et al*. 2018, Eyre *et al*. 2019). Hence, seed application of plant growth promoting microbial inoculants is becoming increasingly used to improve plant health and development at these important early stages (Berninger *et al*. 2018).

During germination, the seed releases carbon (C)- and nitrogen (N)-rich exudates into the spermosphere (Schiltz *et al*. 2015). Moreover, the seed contains phosphorus (P) reserves (predominantly as phytate) contributing to the P nutrition of the young seedling (White and Veneklaas 2012). These P reserves may also become available for seed-associated microbiota, i.e. the microorganisms in the seed, on the seed and in the surrounding spermosphere. Thus, the seed habitat represents a nutrient-rich battle-field, where competition takes place between microorganisms coming with the seed, native soil microorganisms and, when applied, introduced microbial inoculants (Nelson 2004).

Studies of seed-associated bacterial communities have primarily been carried out in axenic or well-controlled soil conditions (Barret *et al*. 2015, Yang *et al*. 2017, Torres-Cortés *et al*. 2018) revealing a dominance of Proteobacteria, Actinobacteria and Firmicutes across several plant species (Nelson *et al*. 2018). Several taxa including *Paenibacillus*, *Pantoea*, *Pseudomonas* and *Xanthomonas* can be transmitted with the seed as endophytes (Links *et al*. 2014, Yang *et al*. 2017, Nelson *et al*. 2018) and some of these can be beneficial to plant growth due to their antagonism against seed-transmitted fungal pathogens (Links *et al*. 2014). Seed-associated bacteria may also be recruited from the soil and later be part of the rhizosphere communities (Johnston-Monje *et al*. 2016). However, insight into the composition and temporal dynamics of seed-associated bacterial communities under realistic field conditions is currently lacking.

In agricultural systems, mineral and organic fertilizers are applied to ensure and improve nutrient availability to crops. Organic fertilizers such as animal manure, can increase both nutrient and organic carbon content in the soil, enhance microbial activity and typically raise soil pH (Zhong *et al*. 2010, Li *et al*. 2015, Blanchet *et al*. 2016). In contrast, amendment with inorganic fertilizers increases nutrient availability, while it typically decreases soil pH either due to oxidation of ammonium-based compounds or due to hydrolysis of orthophosphoric acids (Zhao *et al*. 2014). It is well described that long-term fertilizer amendments have large effects on the diversity and composition of soil bacterial communities (Francioli *et al*. 2016, van der Bom *et al*. 2018), while it remains unknown whether they are equally important in shaping seed-associated communities and their functional potential in relation to nutrient cycling.

Wheat (*Triticum aestivum*) is a stable crop for human diet with an increasing world-wide demand (Shewry and Hey 2015). However, grain yield is stagnating in many regions of the world and the application of chemical fertilizers is not always sustainable. Consequently, there is currently a considerable focus on developing sustainable, plant beneficial microbiological solutions for this crop so the gap between demand and production can be filled.

Although the inoculation to agricultural soils of free-living microorganisms often seems to cause only minor impact on rhizosphere community structure (Ambrosini *et al*. 2016, Silva *et al*. 2021), their impact on the native seed microbiota composition and functional potential in the field remains largely unknown (Ambrosini *et al*. 2016). Using the fungal phosphate solubilizing biofertilizer *Penicillium bilaiae* (Asea *et al*. 1988, Kucey 1988, Kucey and Leggett 1989, Wakelin *et al*. 2004, Leggett *et al*. 2015) and two recently isolated *P. bilaiae* hyphae-associated *Bacillus simplex* strains, shown to stimulate fungal growth and P solubilization *in vitro* (Ghodsalavi 2016, Ghodsalavi *et al*. 2017), as model inoculants, the aims of the current field study were to (i) determine the composition of winter wheat seed-associated bacterial communities as affected by time and by long-term soil amendments with mineral or mineral plus organic fertilizers, (ii) analyze the N and P cycling potential of the bacterial communities as affected by time and soil fertility level, (iii) determine the establishment and persistence of *P. bilaiae* alone and together with *B. simplex*, and (iv) assess the impact of the added inoculants on the indigenous bacterial communities. We hypothesized that (i) time affected the composition and functionality of seed-associated microbiota more than the legacy effect of long-term soil fertilizer amendments, as the exudation from the seed during germination changes substantially over time, and (ii) the added inoculants persisted in the seed-associated community over the entire sampling period.

## Materials and methods

### Field site description

The current field study was conducted at the Long-Term Nutrient Depletion Trial (LTNDT) field established in 1964 at the Experimental Research Farm of the University of Copenhagen in Taastrup, Denmark (55°40′N, 12°17′E). The experimental history, design and management practices have been described in detail by van der Bom *et al*. (2018). Briefly, the entire field received no P or potassium (K), but moderate N fertilizer during 1964–1995, and then the current LTNDT design consisting of seven different fertilizer treatments was established in 1996 (and expanded to 14 treatments in 2010 as described in van der Bom *et al*. 2018), each represented by four replicate plots (50 m × 20 m) in a block design (Fig. S1, Supporting Information). The current study, involved three soil fertility levels: two fully mineral fertilizer (as calcium-ammonium nitrate, triple-superphosphate and potassium chloride, all relatively soluble fertilizers) amendments: (i) N_1_K_1_ (120 kg N, 0 kg P, 120 kg K ha^–1^ y^–1^) that corresponded to a very low P fertility level; (ii) N_1_P_2_K_2_ (120 kg N, 40 kg P, 240 kg K ha^–1^ y^–1^) that resembled a medium P fertility level, a common Danish agricultural practice in medium P and K soils; and one mixed mineral plus organic fertilizer amendment: (iii) M_1_P_1_ (equivalent to an average 120 kg NH_4_-N and 20 kg P and 100 kg K in animal slurry + 20 kg mineral P ha^–1^ y^–1^) resembling a medium to high P fertility level with organic amendment, corresponding to the common application rate for animal slurry in Danish agriculture with supplemental mineral P fertilization. Animal slurry and mineral fertilizer is applied every year during spring. In our experimental period in September—October 2016, no fertilizers were applied right before sowing the winter wheat crop studied, but the preceding crop received animal slurry and first dose of the mineral fertilizer (half the N, all P and K) in April 2016 and the second dose of mineral fertilizer (other half of N) in May 2016; however, soluble nutrients from these were completely depleted in the soil by the time of harvest of the preceding crop and the subsequent sowing of the winter wheat. The soil was a sandy loam with 164 g kg^–1^ clay, 173 g kg^–1^ silt, 333 g kg^–1^ fine sand, 312 g kg^–1^ coarse sand and 17 g kg^–1^ organic matter. The chemical properties of the soils with the selected long-term fertilizer treatments are shown in Table 1.

### Microbial inoculants and experimental design

The microbial inoculants tested in this experiment were (i) the fungus *Penicillium bilaiae* strain DBS5, provided by Novozymes A/S (Denmark) and selected for its ability to solubilize poorly soluble P sources (Raymond *et al*. 2018) and (ii) the *Bacillus simplex* strains 313 and 371, originally isolated from hyphae of *P. bilaiae* strain ATCC 20851, and shown to stimulate growth and P solubilization of *P. bilaiae* under laboratory conditions (Ghodsalavi 2016) and improve P uptake of wheat in a pot experiment (Hansen *et al*. 2020).

In each of the four 50 m × 20 m replicate plots representing the selected soil fertility levels (N_1_K_1_, N_1_P_2_K_2_ and M_1_P_1_), we established four mini-plots (10 m × 3 m) for testing the different microbial inoculants individually and in combination in the following treatments: (i) noninoculated control (C), (ii) single inoculation with *P. bilaiae* (PB), (iii) single inoculation with *B. simplex* strains 313 and 371 (BS) and (iv) combined inoculation with *P. bilaiae* and *B. simplex* strains 313 and 371 (PB+BS). All mini-plots were randomized within each soil fertility level. This resulted in 48 mini-plots (3 soil fertility levels, 4 replicate plots per soil fertility level, and 4 mini-plots per replicate plot, one for each of the inoculum treatments).

## Introduction of microbial inoculants

Microbial inoculation was performed by coating the winter wheat seeds (var. Benchmark) with *P. bilaiae* spores and/or liquid fermentations of *B. simplex* prior to sowing. Briefly, seeds were coated with the dry spores of *P. bilaiae* in a formulation containing 20 g of *P. bilaiae* spores (3.9 × 10^10^ spores g^–1^) and 180 mL of a carrier solution consisting of 66.54% (w/w) sterile water, 0.10% K_2_HPO_4_, 0.02% KH_2_PO_4_, 21.67% maltodextrin and 11.67% maltose monohydrate. The formulation was added to 20 kg of wheat seeds in a compulsory mixer (Soroto maskiner Aps, Glostrup, Denmark) and mixed for 15 min. For the *B. simplex* treatment, 200 mL of a liquid solution consisting of *B. simplex* strains 313 and 371 spores resuspended in carrier solution (total concentration of 1.4 × 10^7^ spores mL^–1^; 0.7 × 10^7^ spores mL^–1^ of each strain) was added to 20 kg of wheat seeds in a compulsory mixer as earlier. For the combined treatment, the used inoculation doses were the same as for the single treatments. The inoculation was done combining the procedures described earlier for the individual strains. For the noninoculated control treatment, 200 mL of carrier solution were added to 20 kg of wheat seeds and coated in the same manner as earlier. After seed treatment, colony-forming units per seed (CFU seed^–1^) were determined by recovering the seed organisms by shaking the seeds in deionized water with 0.1% Tween 80 for 20 min at 250 rpm. Subsequently, dilution series were plated on tryptone yeast agar with 10 mg L^–1^ nystatin (for treatments with *B. simplex*) and potato dextrose agar with 10 mg L^–1^ of streptomycin and penicillin (for treatments with *P. bilaiae*). The control treatment was plated in both media to identify the seed natural occurring community. Determined values were as follows: control, 1.68 × 10^4^ bacterial CFU seed^–1^ + 3.30 × 10^2^ fungal CFU seed^–1^; *P. bilaiae* treatment, 1.83 × 10^5^ CFU seed^–1^; *B. simplex* treatment, 8.13 × 10^4^ CFU seed^–1^; and combined treatment, 1.02 × 10^5^*P. bilaiae* CFU seed^–1^ + 7.96 × 10^4^*B. simplex* CFU seed^–1^. The presented values reflect the natural occurring community on seed for the control samples and the actual size of the inocula on the seeds for the treatments with *B. simplex* and *P. bilaiae* as no other microbial colonies were detected on the plates for those samples (plate pictures in Fig. S2, Supporting Information). The inoculated as well as noninoculated seeds were sown with an experimental sower at a seeding rate of 170 kg ha^–1^ in mid-September 2016, and subsequently managed according to conventional practices.

### Sampling and DNA extraction

Samples of bulk soil were collected 3 days before sowing (DBS). Briefly, from each of the 48 mini-plots, 25–30 soil cores were collected from the plough layer (0–20 cm) following a ‘W’-shaped sampling pattern, and pooled and mixed to one composite sample for each mini-plot. The 48 bulk soil samples were processed for DNA extraction as described later for the seed samples. Furthermore, samples of coated seeds (*n* = 5 per inoculant treatment; total 20 samples) were collected prior to planting and processed as described later.

After planting, seed samples were collected for as long as the seed could be clearly discerned in the field, i.e. 11, 14, 17 and 31 days after sowing (DAS). At each sampling day, 48 samples were recovered (corresponding to three soil fertility levels, four inoculation treatments and four replicates), resulting in 192 samples in total. Briefly, for each sample, 10–12 seedlings were randomly selected and gently removed from the soil with the help of a shovel. Loosely adhered soil was carefully removed from the seedlings by shaking and manually disrupting bigger attached soil aggregates until only closely adhered soil, was left. Shoots and roots were cut off and discarded while the seeds, turning into seed remains during the time of sampling, were collected into a composite sample. Hence, each sample consisted of 10–12 seeds/seed remains with closely adhered soil, comprising the microorganisms in the seed/seed remains, on the seed/seed remains and in the surrounding spermosphere, i.e. soil closely adhered to the seed/seed remains.

The collected samples were freeze-dried for 24 h and finely crushed using zirconium oxide grinding beads or a mortar and pestle prior to DNA extraction. Samples of 0.5 g were used for DNA extraction using the NucleoSpin^®^ 96 Soil kit (Macherey-Nagel, Düren, Germany) adapted to a Biomek^®^ FCP Laboratory Automation Workstation (Beckman Coulter, Brea, CA, USA). Negative controls (500 µL of PCR graded water) were included in the extraction rounds and used for 16S rRNA gene amplicon library preparation together with the seed samples. All extracts were quantified using a Qubit 3.0 fluorometer (Invitrogen, Life Technologies, Nærum, Denmark) with a Qubit^®^ dsDNA HS Assay Kit (range 0.2–100 ng; Invitrogen) and stored at –20°C. DNA concentrations of soil and seed extracts ranged from 35 to 111 ng µL^–1^.

Shoot and root biomass samples were collected once during the study period (17 DAS) to determine if inoculants had any effect on early development of winter wheat. Whole plants with shoots and roots were excavated using a spade from two 0.5-m long rows at different places in each block and combined to obtain one composite sample per plot. The shoots were separated from the roots at the crown, and the roots gently washed under running water. Shoots and root samples were dried in an oven for 48 h at 60°C to determine dry biomass.

## 16S rRNA gene sequencing and bioinformatics

16S rRNA amplicon libraries were prepared from the extracted DNA using the primers 341F (5′CCTACGGGNGGCWGCAG-3′) and 805R (5′GACTACHVGGGTATCTAATCC-3′) with adapters, targeting the V3-V4 regions of the 16S rRNA gene (Yu *et al*. 2005). Detailed library preparation protocol can be found in the Supporting Information. The extracted DNA from the 20 samples of coated seeds were sequenced in an independent run. Paired-end library sequencing was performed using the MiSeq reagent kit v3 (600 cycles) and a MiSeq sequencer (Illumina Inc., San Diego, CA, USA).

The obtained 16S rRNA gene sequences were processed using the UPARSE bioinformatics pipeline version 10.0.240_i86linux64 (Edgar 2013). The paired-end forward and reverse reads were merged using the *-fastq*_*mergepairs* followed by trimming 16 base pairs of the left and 21 base pairs of the right end, corresponding to the PCR primer sequences using *-fastq_truncate* (*-stripleft* 16 *-stripright* 21). Quality filtering was performed using *-fastq_filter* with a maximum expected error of 1 (*-fastq_maxee* 1). All amplicons were reduced to unique sequences using *-fastx_uniques* and counted (*-sizeout*). To recover correct biological sequences, Zero-radius OTUs (zOTUs) were generated with *-unoise3*, removing chimeras and reads with sequencing and PCR errors. Reads with sequencing and PCR errors were remapped to the zOTUs using *-usearch_global* with a minimum sequence similarity of 97% (*-id* 0.97) mapping to the plus strand only (*-strand* plus). The taxonomic classification of the zOTUs was conducted using QIIME2 2019 v. 10 (Bolyen *et al*. 2019) and classify-sklearn and the SILVA database v. 3 (Quast *et al*. 2012). zOTUs classified as Mitochondria or Chloroplast were removed from the dataset. Furthermore, zOTUs classified as Unknown at kingdom level or unclassified Bacteria at phylum level also represented plant DNA contamination based on BLAST searches and were removed. Archaeal sequences were also removed from the dataset. The raw sequences were uploaded to NCBI Sequence Read Archive (SRA) under the bioproject number PRJNA649549. The number of sequences before and after cleanup, the final number of zOTUs, and the individual SRA sample accession numbers are presented in Table S1 (Supporting Information).

### Functional gene qPCR array

A panel of 30 bacterial and archaeal functional genes involved in cycling of N or P, as well as the 16S rRNA gene were quantified in parallel by a high-throughput qPCR (HT-qPCR) quantitative microbial element cycling (QMEC) chip employing a SmartChip real-time PCR system (WaferGen Biosystems, Fremont, CA, USA) (Zheng *et al*. 2018) (primer sequences can be found in Table S2, Supporting Information). The thermal program was as follows: 10 min at 95°C,  followed by 40 cycles of 30 s at 95°C, 30 s at 58°C and 30 s at 72°C. The WaferGen software automatically generated melting curves. Three technical triplicates were included in HT-qPCR, and only samples showing positive amplification of all technical triplicates was considered for further data analysis. Gene copy number was calculated using the equation: gene copy number = 10 ^((31-Ct)/(10/3))^, where Ct is the threshold cycle (Looft *et al*. 2012). Functional genes were normalized to the amount of 16S rRNA genes to obtain relative abundances (copy numbers of functional genes per 16S rRNA gene). Water samples were included as negative controls.

### Microbial inoculant persistence

Quantification of *P. bilaiae* was performed by digital droplet PCR (ddPCR). ddPCR reactions were prepared in 30 μL volumes using 15 µL of QX200 EvaGreen ddPCR Supermix (Bio-Rad Laboratories Inc., CA, USA), 0.1/0.25 μM of each primer (-ITS-F2: 5′-CGCCGAAGCCCCCTCTG-3′; ITS-R: 5′-GCATTTCGCTGCGTTCTTCA-3′; Gómez-Muñoz *et al*. 2017), 10.5 µL of PCR graded water and 1.5 μL of DNA extracted from the seed samples. For negative controls, 1.5 μL of water was added instead of DNA. Positive controls were carried out by using a dilution series of genomic DNA extracted from pure cultures of *P. bilaiae*.

Twenty microliters of the reaction mixture were loaded into a sample well of a DG8 cartridge (Bio-Rad Laboratories Inc., CA, USA), while 70 μL of Droplet Generation Oil for EvaGreen^®^ (Bio-Rad Laboratories) were loaded in the corresponding oil well. The cartridge was transferred to a QX200 Droplet Generator (Bio-Rad Laboratories) to generate up to 20 000 droplets, according to the manufacturer's instructions. The emulsion was then gently transferred to a twin.tec semi-skirted 96-well PCR plate (Eppendorf, Hamburg, Germany), which was sealed with Pierceable Foil Heat Seal (Bio-Rad Laboratories) using a PX1 PCR Plate Sealer (Bio-Rad Laboratories). The plate was immediately put in a T100 Thermal Cycler (Bio-Rad Laboratories) where end-point amplification was performed under the following conditions: initial denaturation at 95°C for 3 min, followed by 5 cycles touchdown starting at 64.6°C and decreasing by 0.4°C per cycle, after which 25 cycles of 30 s at 95°C, 30 s at 63°C, 30 s at 72°C were carried out before a final extension for 7 min at 72°C. After completion of the PCR, the sealed plates were moved into the QX200 Droplet Reader (Bio-Rad Laboratories) where droplets and respective signals were analyzed according to manufacturer's recommendations.

For *B. simplex* strains 317 plus 371, their joint persistence on the seeds was assessed using the 16S rRNA gene amplicon data by comparing the relative abundance of the zOTU 22 in control and inoculant treatments. The relative abundance of the *Bacillus* zOTU 22 was used as a proxy for persistence of these two strains in the experiment as the genomic similarity between the two *Bacillus simplex* strains used in this experiment is not sufficient to be able to distinguish them using the V3-V4 regions of the 16S rRNA gene.

### Data analysis and statistics

All statistical analyses were performed with R version 3.6.1 (R Core Team 2019) and making substantial use of the phyloseq (McMurdie and Holmes 2013), vegan (Oksanen *et al*. 2018) and ggplot2 (Wickham 2016) packages. For the sequence data, rarefaction curves at zOTU level were computed using the vegan package (Oksanen *et al*. 2018). The rarefaction curves indicated that the number of zOTUs increased with the number of sequences although they did not reach a true plateau (Fig. S3, Supporting Information). Hence, a deeper sequencing effort is needed to fully cover the diversity of the bacterial communities in the current environment. The *rrarefy* function was used to rarefy the generated annotation tables to 2000 reads per sample, and diversity indices (Richness (S = number of different zOTUs) and Shannon's ($H = - \sum\nolimits_{i = 1}^R {{p_i}\ln {p_i}} $) at zOTU level were calculated using the phyloseq package (McMurdie and Holmes 2013). Differences in the diversity indices of the seed-associated samples (collected at 11, 14, 17 and 31 DAS) were determined using three-way ANOVA. Post-hoc tests were then done using the *emmeans* package v. 1.7.2. (Lenth 2022) on adjusted linear models using the identified significant factors. *P* values were adjusted using the Tukey honest significant difference (HSD) test and considered significant when <0.05. Diversity of bulk soil samples collected at 3 DBS was plotted side by side with the seed-associated samples for reference. Downstream analyses were performed using a non-rarefied dataset where samples with <1000 reads were removed. The exception to this were the 20 seed samples collected prior to sowing that harbored a very limited microbial community and a high plant DNA content leading to <1000 bacterial reads per sample, corresponding to 1–27 zOTUs per sample (Table S1, Supporting Information). Samples with <10 reads were removed from the downstream analysis. The analysis of those samples was therefore done using non-rarefied data without the >1000 reads threshold. The low number of reads does not allow for a complete characterization of the initial seed microbiome, as several taxa have not been detected. However, by sequencing the coated seed samples independently of the rest of the samples, we avoided risk of cross-contamination and index-hopping. Hence, we argue that the detection of specific zOTUs in these samples highlight their presence in the initial seed community, although we cannot make any inferences on relative abundance. Beta-diversity at zOTU level was represented by nonmetric multidimensional scaling (NMDS) ordination using Bray–Curtis dissimilarities on relative abundances. In parallel, PERMANOVA (1000 permutations; Bray–Curtis dissimilarity index) was used to evaluate the effects of soil fertility level, time and inoculation treatment to the seed-associated samples (collected at 11, 14, 17 and 31 DAS).

The differential abundance of genera between inoculant treatments was analyzed, while controlling for soil fertility level at the four sampling days. The differential abundance of classes and phyla between sampling times were analyzed for each soil fertility level, while controlling for inoculation treatment. The differential abundance was determined using beta-binomial regression with the Corncob package v.1.0 (Martin *et al*. 2020). Only genera that had an estimated differential abundance of < –1 or >1, and *P*-values adjusted for multiple testing <0.05 (FDR < 0.05) were considered significant.

For the analysis of the functional genes, only genes that were above the detection limit in three out of four biological replicates were admitted into the analysis. This excluded the genes *hzo*,*bpp* and *hzsA*. The relative abundances of functional genes in different conditions were compared using Bray–Curtis dissimilarities. PERMANOVA (1000 permutations, Bray–Curtis dissimilarity index) was used to test for the effect of soil fertility level, inoculation treatment and time on the composition of the functional potential. Differences in relative abundance at 11 and 31 DAS were tested using a Mann–Whitney test as the data did not fit a normal distribution. Obtained *P*-values were corrected using the Benjamini–Hochberg correction to account for multiple testing. Differences with adjusted *P*-values < 0.01 were considered significant.

For the analysis of the microbial inoculant persistence, two-way ANOVA was used to determine the effect of soil fertility level and inoculation treatment within each time point, for both *P. bilaiae* ddPCR data and for *Bacillus* strain 313+317 (both included in zOTU 22) relative abundance data. For the analysis of shoot and root biomass, two-way ANOVA was used to determine the effect of inoculation treatments and soil fertility level. A linear mixed model was used to account for both fixed and random field block effects. Differences with *P*-values < 0.05 were considered significant.

## Results

### Seed development and climatic data

Winter wheat with different inoculant treatments was sown in plots with different soil fertility levels. Independent of treatment or soil fertility, seeds germinated 8 DAS. Seeds with closely adhered soil were subsequently sampled from 3 to 23 days after germination, i.e. 11, 14, 17 and 31 DAS. During the 31 days of sampling, the seeds slowly decomposed. We refer to bacteria recovered from these samples as seed-associated later. During this time period, the plants developed to reach Zadock growth stage 12 (the two-leaf stage) across all soil fertility levels. The average temperature dropped from ∼15°C to ∼10°C and the site received ∼85 mm of precipitation during this period (Fig. S4, Supporting Information). The microbial inoculation had no effect on early shoot or root biomass of winter wheat as measured at 17 DAS (Fig. S5, Supporting Information). The highest shoot biomass was achieved at N1P2K2 fertility level, the root biomass was equally high at N1K1 and N11P2K2 fertility levels. While the plant parameters are of high importance for evaluating inoculant performance, the main focus of the present study was the development of the seed-associated bacterial community as affected by inoculants and soil fertility levels.

### Diversity and community structure are affected more by time than by soil fertility level and inoculation treatments

**Figure 1.**
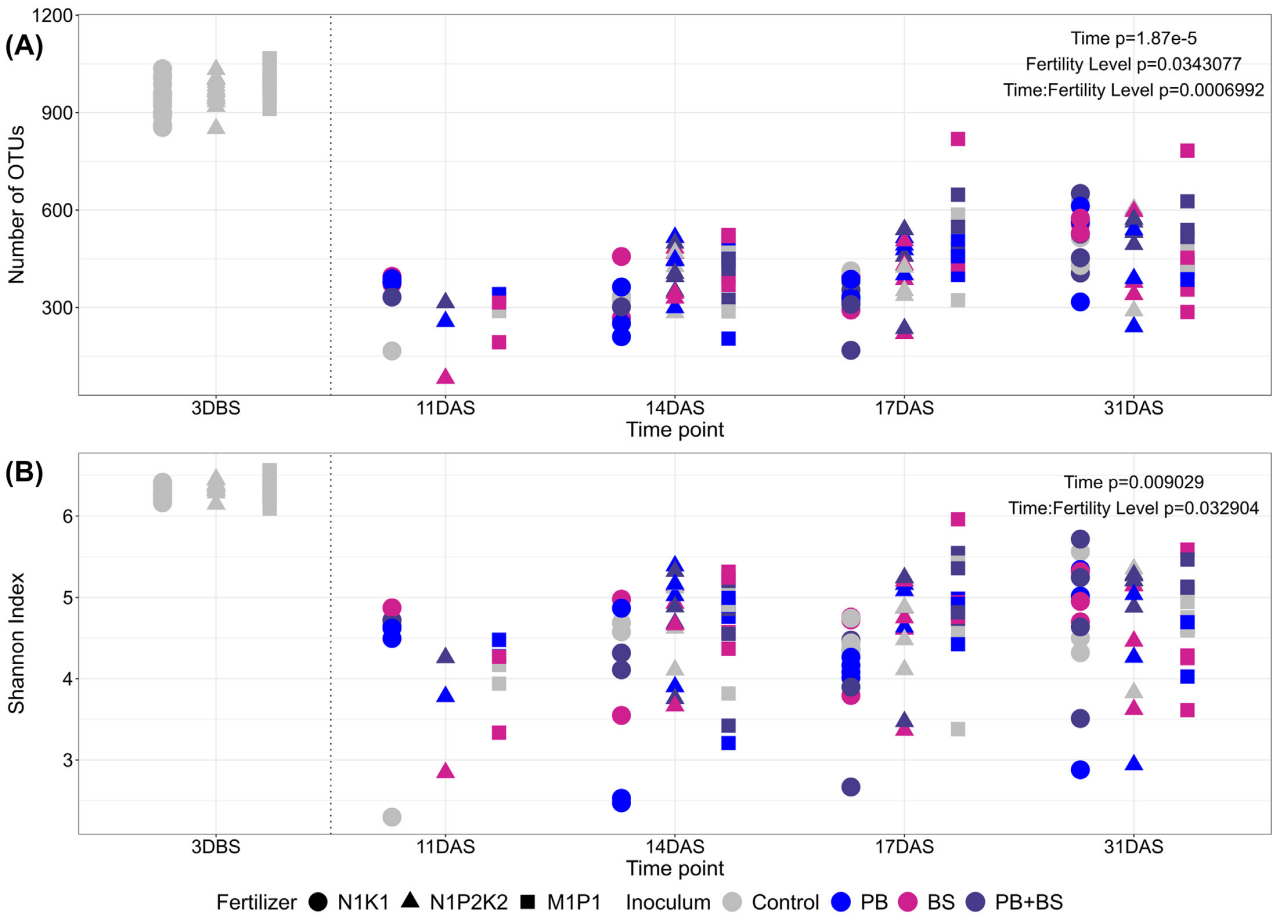
Diversity indices calculated from 16S rRNA gene sequence data at zOTU level for soil at 3 days before sowing, and for seed-associated samples at 11, 14, 17 and 31 days after sowing and for each soil fertility level (5 < *n* < 16 except for N_1_P_2_K_2_ at 11 DAS where *n* = 3): **(A)** Richness index (number of zOTUs) and **(B)** Shannon diversity index. Samples were rarefied to 2000 reads prior to analysis. Each sample is represented by a point where color identifies the applied inoculum and shape the fertility level where it was collected from. For the seed-associated samples, factors showing a significant impact to each diversity index (ANOVA followed by post-hoc tests using the Tukey method; *P*< 0.05) are depicted in the respective panel with the corresponding *P* values. DBS: days before sowing; DAS: days after sowing.

At 3 DBS, the Richness and the Shannon diversity for the soil bacterial communities were significantly higher than for the seed-associated bacterial communities at each time point (Fig. 1; *P* < 0.001). However, the Richness and the Shannon diversity increased significantly with time for the seed-associated communities (Fig. 1). Moreover, a significant interaction between time and soil fertility level was noted for both the Richness and the Shannon diversity (Fig. 1). For M1P1 and N1P2K2, the Richness was significantly lower at 11 DAS compared with the following sampling time points (Tukey HSD; N1P2K2: *P* < 0.01; M1P1: *P* < 0.05), whereas 11, 14 and 17 DAS were significantly lower than 31 DAS for N1K1 (Tukey HSD; *P* < 0.001). The Shannon diversity was significantly higher at 31 DAS compared with 14 and 17 DAS for N1K1 (Tukey HSD; *P* < 0.05). No differences in Shannon diversity were observed between sampling time points for M1P1. Among fertility levels, Richness and Shannon diversity were significantly higher forM1P1 and N1P2K2 than N1K1 at 14 DAS (Tukey HSD, Richness: *P* < 0.05; Shannon Diversity: *P* < 0.05). At 17 DAS, the Richness differed significantly among the three fertility levels (M1P1 > N1P2K2 > N1K1; Tukey HSD; *P* < 0.05) and between two fertility levels for the Shannon diversity (M1P1 > N1K1; Tukey HSD; *P* = 0.0121). There were no significant effects of the introduced inoculants *P. bilaiae*, *B. simplex* or the combination of *P. bilaiae* and *B. simplex* on the bacterial community alpha diversity.

**Figure 2.**
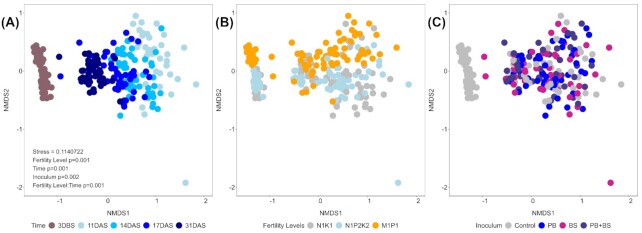
NMDS ordination plots of Bray–Curtis dissimilarities between soil and seed-associated bacterial communities, colored according to time **(A)**, soil fertility level **(B)** or applied inoculum **(C)**. The data were obtained by sequencing of the v3–v4 regions of the 16S rRNA gene. Only factors significantly affecting the structure of the community of the seed-associated samples (PERMANOVA with 1000 permutations; *P* < 0.05) are depicted in the chart followed by the corresponding *P* values. DBS: days before sowing; DAS: days after sowing.

The compositions of soil and seed-associated bacterial communities were significantly different (Fig. 2). For the seed-associated communities, their composition was significantly affected by the tested factors time, soil fertility level and inoculum (Fig. 2) (PERMANOVA; *P* = 0.001 for Time and Fertility Level; *P* = 0.002 for Inoculum; Table S3, Supporting Information). Time was the most prominent predictor of the community composition (*R*^2^ = 0.27). Accordingly, the sample clustering indicated that the bacterial community changed gradually from 11 until 31 DAS and became more similar to the soil community (Fig. 2A). While soil fertility level was also significant, it explained less of the variation (Fig. 2B) (*R*^2^ = 0.036). Moreover, the interaction of these two factors also significantly influenced the community composition showing that soil fertility level shaped the community within each time point (*R*^2^ = 0.065). The effect of the introduced inoculants was also significant, but this factor was the poorest predictor of community composition (Fig. 2C) (*R*^2^ = 0.025). Thus, the introduction of inoculants had low impact on the seed-associated community structure compared with time and soil fertility level.

**Figure 3.**
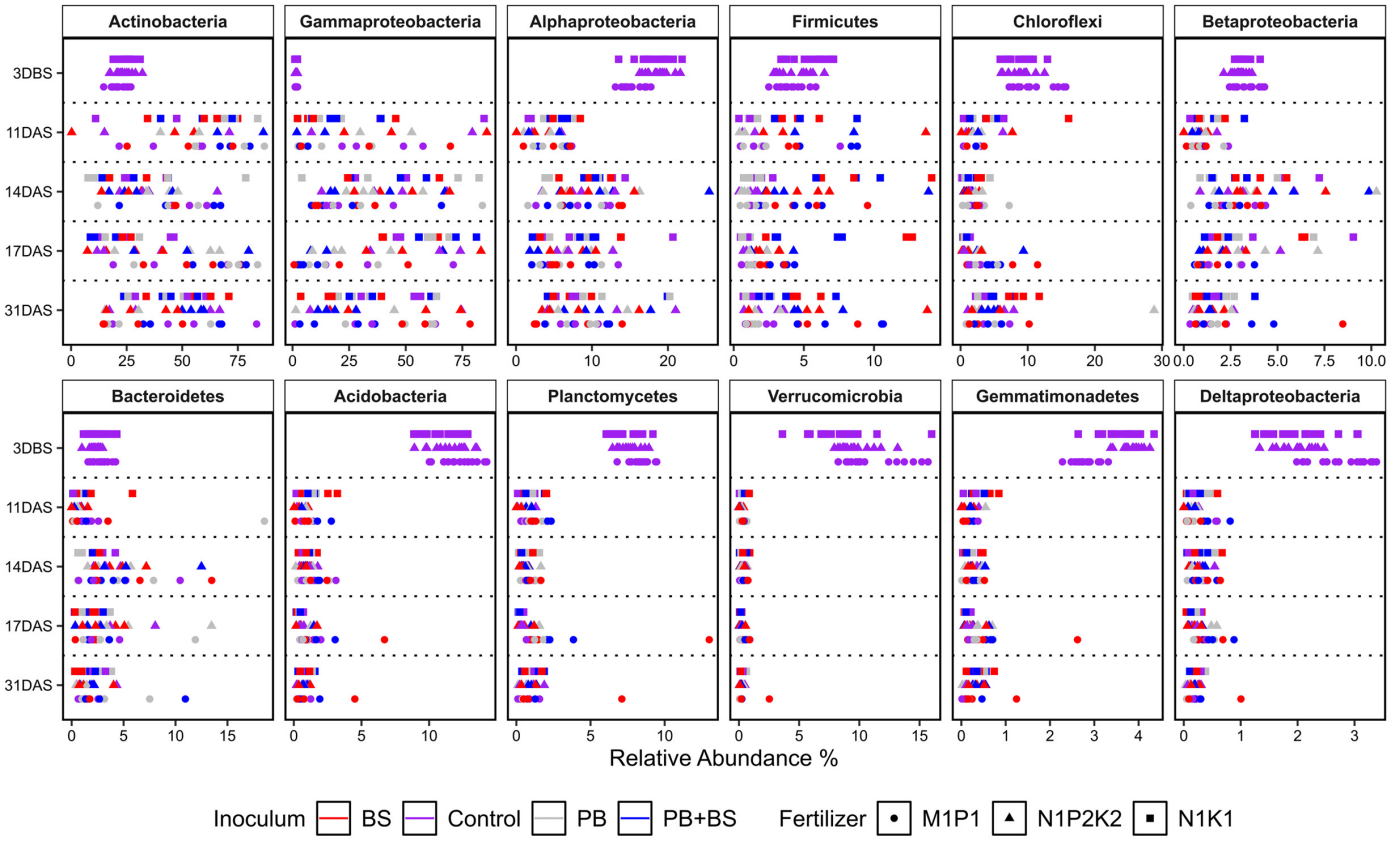
Relative abundance of the 12 most abundant taxa present at each soil fertility level at each sampling time point (5 < *n* < 16, with the exception of N_1_P_2_K_2_ at 11 DAS where *n* = 3). Data are presented at the phylum level, with the exception of Proteobacteria, which are classified to class level. Each sample is represented by a point where color identifies the applied inoculum and shape the fertility level where it was collected from. DBS: days before sowing; DAS: days after sowing.

The communities at 11 DAS were dominated by Actinobacteria and Gammaproteobacteria for all soil fertility levels (Fig. 3). In contrast, the soil at 3 DBS was dominated by Actinobacteria, Acidobacteria, Alphaproteobacteria, Chloroflexi and Verrucomicrobia. At the N_1_K_1_ soil fertility level, the Gammaproteobacteria significantly increased their relative abundance between 11 and 17 DAS (FDR < 0.01), after which the relative abundance decreased again to the level observed at 11 DAS. Their temporal dynamics coincided with a significant decrease in the relative abundance of Actinobacteria from 11 DAS to 14 and 17 DAS (FDR < 0.05), after which the Actinobacteria again increased in relative abundance at 31 DAS. For the N_1_P_2_K_2_ soil fertility level, the dynamics in Actinobacteria and Gammaproteobacteria resembled those in N_1_K_1_, although the changes were smaller and not statistically significant. Considering the M_1_P_1_ fertility level, the Actinobacteria showed relative abundances over 50% at 11, 14 and 17 DAS, and then showed a significant decline to ∼35% (FDR = 0.01). This again coincided with an increase in Gammaproteobacteria between 17 and 31 DAS. Among the less abundant taxa, the Alphaproteobacteria showed a significant increase in relative abundance after 11 DAS for the N_1_K_1_ (FDR < 0.001) and N_1_P_2_K_2_ fertility levels (FDR < 0.005). Furthermore, the relative abundance of Bacteroidetes was significantly higher, as compared with 11 DAS, at 14, 17 and 31 DAS or at 14 DAS for the N_1_P_2_K_2_ and M_1_P_1_ fertility levels, respectively; while the Betaproteobacteria showed the significantly highest relative abundances at 14 and 17 DAS across soil fertility levels (FDR < 0.05) (Fig. 3).

When the impact of the inoculation treatments was determined at the genus level for individual days, the genus *Bacillus* had significantly higher relative abundance in treatments with *B. simplex* and *P. bilaiae* plus *B. simplex* (Fig. S6, Supporting Information). No other genera were consistently significantly enriched or reduced at all sampling times (Fig. S6, Supporting Information). At the family level, the seed-associated microbiomes were dominated by Enterobacteriaceae, Micrococcaceae, Pseudomonadaceae, Streptomycetaceae, and Infrasporangiaceae across time, soil fertility level and inoculation (Fig. S7, Supporting Information).

**Figure 4.**
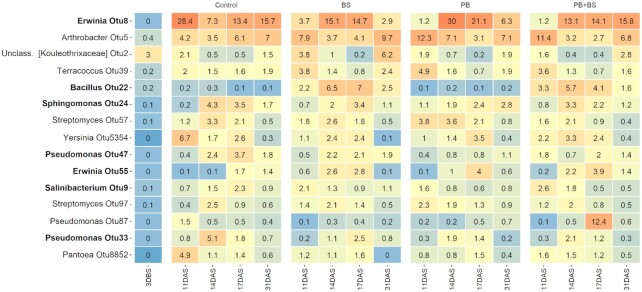
Relative abundance of the 15 most abundant zOTUs across sampling times of samples from the N1K1 soil fertility level. Relative abundances are mean values across sampling times. Three days before sowing (DBS) bulk soil samples (*n* = 48), and 11, 14, 17 and 31 days after sowing (DAS) (*n* = 4). zOTUs detected in original seed samples (seed samples prior to sowing and germination) are highlighted in bold. *Bacillus* OTU22 were only detected in seeds inoculated with *B. simplex* (BS and PB+BS).

Furthermore, the 15 most abundant zOTUS across seed-associated communities at 11–31 DAS and the soil community at 3 DBS were determined (Fig. 4; Fig. S8, Supporting Information). The most abundant zOTU belonged to the genus *Erwinia* and in general we found high relative abundance of zOTUs belonging to the genera *Arthrobacter*,*Erwinia*, *Pseudomonas*,*Sphingomonas* and *Streptomyces*. Several zOTUs, including some within the genera *Erwinia*, *Pseudomonas*,*Salinibacterium* and *Sphingomonas*, were also detected in seeds prior to germination (Fig. 4). The *Bacillus* OTU22 was only detected in seeds that were coated with the *Bacillus simplex* strains (Fig. 4). Other zOTUs within *Arthrobacter*,*unclassified Kouleothrixaceae*, *Streptomyces* and *Terracoccus* were common for soil and seed-associated communities but not detected on original seeds (Fig. 4).

### *Penicillium bilaiae* and *B. simplex* persist in the seed-associated community until 31 DAS

**Figure 5.**
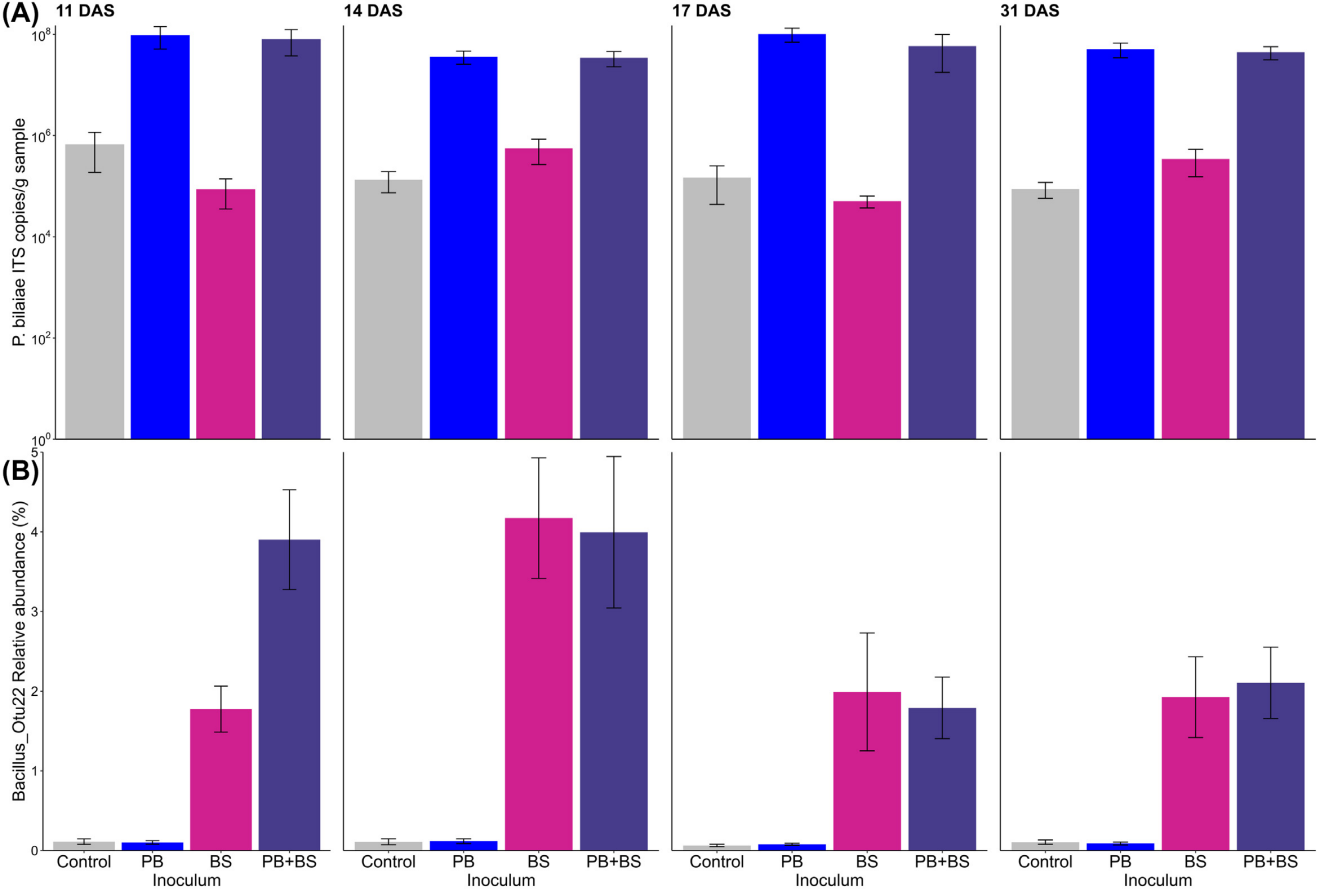
Persistence of the inoculants *Penicillium bilaiae* (PB) and *Bacillus simplex* (BS) on wheat seeds over time. **(A)** Digital droplet PCR-based quantification of the *P. bilaiae* across inoculum treatments and time. The results are presented on a logarithmic scale. Error bars depict standard errors of average values (10 < *n* < 12). **(B)** Relative abundance of zOTUs assigned to the zOTU 22 of the genus *Bacillus* across inoculum treatments and time. Error bars depict standard error of average values (7 < *n* < 12). DAS: days after sowing.

The inoculation treatments that had received *P. bilaiae* alone or in combination with *B. simplex*, had population sizes of *P. bilaiae* of ∼1 × 10^8^ ITS copies g sample^–1^ throughout the sampling period (Fig. 5A). There were no consistent effects of the soil fertility levels on the population size for any sampling day. A significantly lower indigenous population of *P. bilaiae* below 1 × 10^6^ ITS copies g sample^–1^ was found for the samples that had not been inoculated with *P. bilaiae* (*P* < 0.022, except at 11 DAS). The relative abundance of the *Bacillus z*OTU 22 was significantly higher for treatments that had received *B. simplex* inoculants than for control treatments throughout the sampling period (Fig. 5B; *P* < 3 × 10^–5^), indicating persistence of the inoculum. Moreover, a small effect of the soil fertility level was noted for the relative abundance of the *Bacillus* zOTU 22 17 DAS (*P* = 0.001), where values were higher for the N_1_K_1_ fertility level (Fig. 4) compared with N_1_P_2_K_1_ and M_1_P_1_ (Fig. S8, Supporting Information). In summary, regardless of the soil fertility level the *P. bilaiae* and *B. simplex* strains were still found in or on the seeds at 31 DAS.

### The potential of seed-associated communities for N and P cycling changes with time

A panel of functional genes involved in cycling of N and P were quantified using high throughput qPCR, and relative abundances were calculated after normalization with 16S rRNA gene copy numbers. For the relative abundances of these genes, time (*R*^2^ = 0.40) and soil fertility level (*R*^2^ = 0.022) were significant predictors (PERMANOVA, *P* < 0.001 for both factors), while the addition of inoculants had no significant effect (Table S4, Supporting Information). The temporal changes in the relative abundances of the panel of genes occurred gradually between 11 and 31 DAS (Fig. S9, Supporting Information), and we therefore describe the changes between these two time points later. Furthermore, the soil fertility level only affected the relative abundance of very few functional genes, and we consequently describe changes collectively across soil fertility levels later, while making specific references to the genes affected by the soil fertility level.

**Figure 6.**
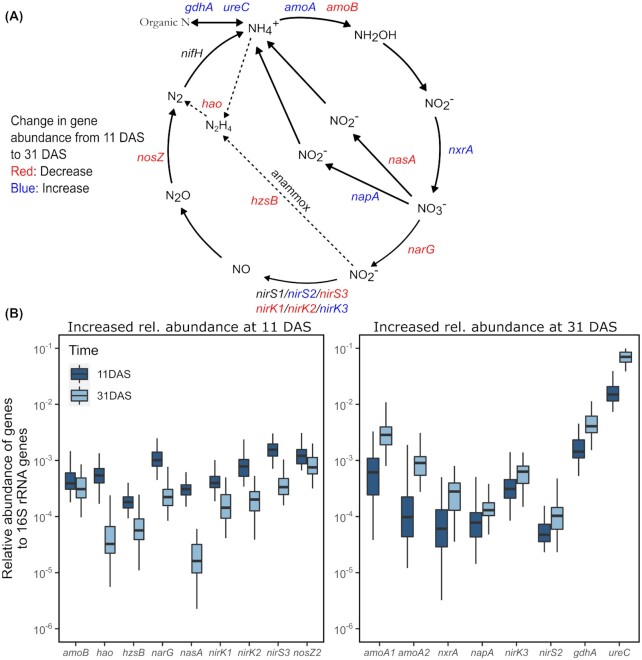
Changes in relative abundance of genes involved in nitrogen cycling. **(A)** Overview of genes targeted by the high-throughput qPCR array. Genes in red decreased in relative abundance and genes in blue increased. Genes in black did not change with time. **(B)** Relative abundances of gene copies normalized to copies of 16S rRNA genes at 11 and 31 days after sowing (DAS) (*n* = 48). Only genes that have significantly different relative abundance between the two sampling times are shown (*P* < 0.01, Mann–Whitney Test). Genes in the left panel had a significantly higher relative abundance at 11 DAS, while genes in the right panel had a significantly higher relative abundance at 31 DAS. The box plots show the median, the two hinges, which correspond to the 25th and 75th percentile, and the upper and lower whiskers, which extend from higher and lower hinges to the largest and smallest values no further than 1.5 times the interquartile range.

Several genes involved in N cycling decreased significantly over time (*P* < 0.01, Mann–Whitney test), showing the highest relative abundance at 11 DAS across all soil fertility levels (Fig. 6). This included genes involved in anaerobic ammonium oxidation (anammox) such as hydroxylamine oxidoreductase (*hao*) and hydrazine synthetase (*hzsB*), as well as aerobic ammonia oxidation (*amoB*). In addition, the nitrate reductases (*nasA* and *narG*) (Fig. 6A) decreased from 11 to 31 DAS in all soil fertility levels, while the nitrous oxide reductase (*nosZ2*) decreased from 11 to 31 DAS only in the N_1_P_2_K_1_ soil fertility level. For the nitrite reductases *nirS* and *nirK*, some variants showed decreased abundance with time, while others showed increased abundance with time (Fig. 6B). The *amoB* gene was significantly lower in the N_1_K_1_ soil fertility level at 31 DAS compared with 11 DAS.

Other genes increased in relative abundance over time, showing the highest relative abundance at 31 DAS (*P* < 0.01, Mann–Whitney test) (Fig. 6B). Among those were the genes encoding enzymes involved in aerobic ammonia oxidation (*amoA*) (both bacterial and archaeal) as well as genes involved in nitrite oxidation (*nxrA*). In addition, the *ureC* and *gdhA* genes involved in organic nitrogen mineralization increased in relative abundance with time. Some of these genes only differed in N_1_K_1_ and M_1_P_1_ (*nirS2*, *napA*), N_1_K_1_ (*nirK3*), or N_1_K_1_ and N_1_P_2_K_1_ soil fertility level (*nxrA*). Only the relative abundance of *nifH* and *nirS1* did not change with time.

**Figure 7.**
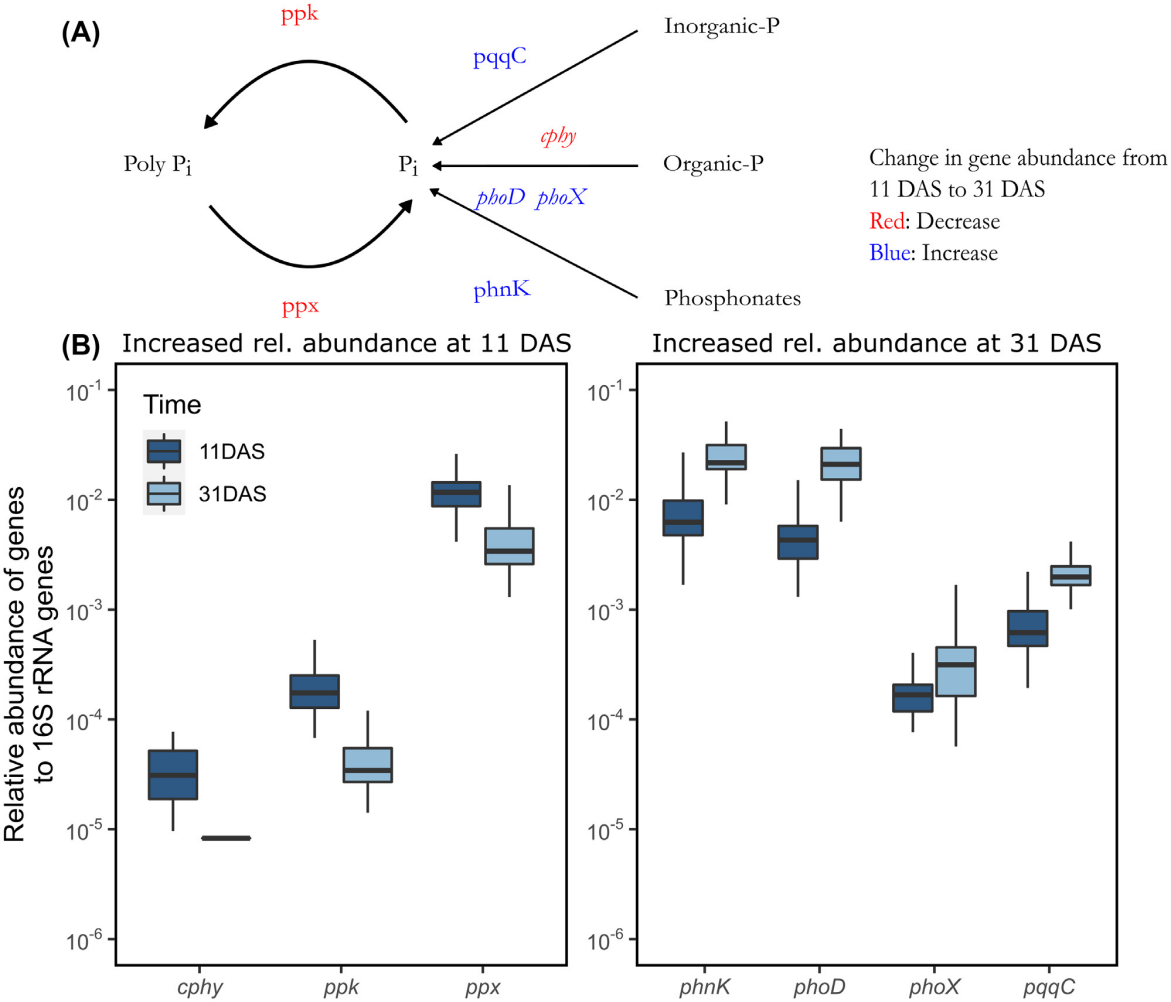
Changes in relative abundance of genes involved in phosphorus cycling. **(A)** Overview of genes targeted by the high-throughput qPCR array. Genes in red decreased in relative abundance and genes in blue increased. **(B)** Relative abundances of gene copies normalized to copies of 16S rRNA genes at 11 and 31 days after sowing (DAS) (*n* = 48). At 31 DAS, *cphy* was below the detection limit for most of the samples. Only genes that have significantly different relative abundance between the two sampling times are shown (*P* < 0.01, Mann–Whitney Test). Genes in the left panel had a significantly higher relative abundance at 11 DAS, while genes in the right panel had a significantly higher relative abundance at 31 DAS. The box plots show the median, the two hinges that correspond to the 25th and 75th percentile, and the upper and lower whiskers, which extend from higher and lower hinges to the largest and smallest values no further than 1.5 times the interquartile range.

For the selected genes involved in P cycling, *ppk* and *ppx*, involved in the formation and degradation of polyphosphate, respectively, were found in significantly higher abundance at 11 DAS (*P* < 0.01, Mann–Whitney test) (Fig. 7). Moreover, the gene coding for cysteine phytase (*cphy*), initiating the hydrolysis of phytate to release phosphate, was only detected in the samples at 11 DAS but not at 31 DAS (Fig. 7B). In contrast, *phoD* and *phoX* genes encoding alkaline phosphatases involved in mineralization of organic P sources, the *phnK* gene involved in utilization of phosphonate, and the *pqqC* gene involved in solubilization of inorganic phosphorus had higher relative abundance at 31 DAS (Fig. 7B). The gene *phoX* were only significantly different between 11 and 31 DAS in the N_1_P_2_K_1_ soil.

## Discussion

### The development of the seed-associated bacterial communities

The seed-associated microbiome is thought to be important for the establishment of the rhizosphere microbiome that has a pivotal role in plant health and yield (Shade *et al*. 2017). However, the development of seed-associated microbiome under field conditions is rarely studied. Here, we provide the first field study addressing the development of wheat seed-associated bacterial communities during seedling emergence and how they are affected by soil fertility levels and inoculants.

The seed-associated communities had lower alpha diversity than the bulk soil samples, in line with findings from maize and *Brassica napus* (Rochefort *et al*. 2021, Shao *et al*. 2021). The seed-associated communities were dominated by Actinobacteria, Gammaproteobacteria and Alphaproteobacteria, while the corresponding bulk soil samples were dominated by Actinobacteria, Acidobacteria, Alphaproteobacteria, Chloroflexi and Verrucomicrobia in agreement with previous reports for these soils (van der Bom *et al*. 2018, Zhang *et al*. 2020). Some of the dominating seed-associated taxa (*Erwinia*, *Pseudomonas*,*Salinibacterium* and *Sphingomonas*) might come with the seeds as endophytes (Özkurt *et al*. 2019, Kuźniar *et al*. 2020) or as epiphytes (Links *et al*. 2014). Several zOTUs were found both in the original seeds and in the seed-associated samples even 31 DAS, in accordance with previous studies. As the seed and seed-associated samples were sequenced in different runs, we can exclude the possibility of cross contamination. Hence, we propose that some seed-associated taxa come from the seeds; although the low number of sequences from the original seeds prevent us from making definite conclusions on their relative abundance in the original seeds. Studies that addressed the epiphytic versus the endophytic bacterial communities of soil-free or surface sterilized wheat seeds found that dominating epiphytic taxa included Proteobacteria (the genera *Massilia*, *Sphingobium*,*Sphingomonas* and *Xanthomonas*; Links *et al*. 2014), while Proteobacteria and Firmicutes (*Acinetobacter*,*Paenibacillus* and *Paracoccus*) dominated in the endosperm, and members of the Enterobacteriaceae and Pseudomonadaceae (*Pantoea* and *Pseudomonas*) were abundant in both compartments (Links *et al*. 2014, Kuźniar *et al*. 2020). However, the current seed-associated communities also contained abundant genera such as *Arthrobacter*,*Kaistobacter*, *Streptomyces*, and *Terracoccus* that were shared with the soil. In addition, the alpha diversity of seed-associated communities increased with time and the communities increasingly resembled soil communities. Thus, our findings support the notion that seed-associated bacterial communities are assembled from both seed and soil-borne taxa. Previous studies have shown that rhizosphere communities include both seed and soil derived taxa (Johnston-Monje *et al*. 2016, Kavamura *et al*. 2019). Interestingly, Walsh *et al*. (2021) recently demonstrated that while seed microbiota contribute significantly to the wheat seedling bacterial community, the influence of soil-derived communities on the seedling microbiome is predictable, yet variable between soils. In our current study, several seed-borne taxa persisted in the seed-associated communities even 31 DAS, while several soil-borne taxa emerged during germination, with the potential to influence the health of the emerging seedling (Nelson 2017).

### The composition and N/P cycling potential of the seed-associated communities are highly dynamic during germination

High concentrations of resources are available at the seed during germination and emergence (Nelson 2004, Schiltz *et al*. 2015, Nelson *et al*. 2018), and the high abundance of copiotrophic genera as *Erwinia*, *Pseudomonas*,*Salinibacterium* and *Sphingomonas* suggest an enrichment in and on the seed of taxa able to degrade seed components and seed exudates (Lemanceau *et al*. 2017). Moreover, seed germination and early seedling development is a dynamic phase of a plant's life cycle (Eyre *et al*. 2019). In accordance, time was the most important factor shaping the composition of the seed-associated bacterial community in this study. The seeds germinated at 8 DAS, and the current sampling period hence covered the period of seed exudation that, under laboratory conditions, lasts a few days after germination (Schiltz *et al*. 2015), but is probably extended at the current ambient temperatures between 10 and 15°C. At the N_1_K_1_ soil fertility level, the Gammaproteobacteria increased in relative abundance with time up to 17 DAS, while the relative abundance of Actinobacteria decreased. For comparison, an increase in Gammaproteobacteria has been recorded during emergence for seeds of *Brassica* species, and a comparable increase concomitant with a decrease in Actinobacteria was seen for bean and radish seeds in laboratory systems (Barret *et al*. 2015, Torres-Cortés *et al*. 2018).

In contrast to time, the soil fertility level had a much smaller effect on the alpha diversity and composition of seed-associated communities. Further, soil fertility level did not have an effect on the alpha diversity of the bulk soil communities (3 DBS), in agreement with previous findings from the same fields (van der Bom *et al*. 2018). Overall, the current data support our first hypothesis that time has a larger effect on the community composition than long-term history of soil fertilizer amendment.

The relative abundances of genes involved in aerobic and anaerobic N cycling, as well as in P cycling, were used as proxies for the processes catalyzed by their predicted proteins. Despite 16S rRNA gene copies varies between bacterial taxa (Větrovský and Baldrian 2013), normalization to 16S rRNA copies is used as a general proxy for bacterial abundance, and hence used in the present study as a mean of comparing across samples. Genes related to anaerobic ammonia oxidation (*hao* and *hzsB*) carried out by strictly anaerobic Planctomycetes (Lage and Bondoso 2014) significantly decreased from 11 to 31 DAS. Anammox bacteria are able to convert organic compounds to sustain their metabolism, most notably formate, acetate and propionate (Kartal *et al*. 2013), which could explain the abundance of these genes in or around the seeds in the beginning of the period, as seed exudates containing organic acids are released during germination and emergence (Nelson 2004, Schiltz *et al*. 2015). In contrast, genes involved in aerobic ammonia oxidation (*amoA*) increased with time. Furthermore, the findings suggest that ammonium was available for the seed-associated communities throughout the sampling period as seen from an increase of genes involved in ammonium-generating organic N transformation (*ghdA* and *ureC*). This increase could reflect ammonification of amino acids and urea from seed exudates (urea has been found in root exudates of other cereals; Naveed *et al*. 2017), or of amino acids coming from degradation of proteins found in cells of the outer layers of the wheat grain (Šramková *et al*. 2009). These findings indicate a higher microbial activity and oxygen consumptions early after germination, supporting a transition from an initial anaerobic environment to aerobic conditions following seed germination. This is in accordance with previous report of high oxygen consumption and at times anaerobic conditions in both the rhizosphere and spermosphere (Højberg and Sørensen 1993, Bewley *et al*. 2013).

For the genes involved in nitrate reduction, *nasA* and *narG* decreased, whereas *napA* was found to increase in abundance. A reduction in genes encoding nitrate reductases would be expected during a period with increased oxygen levels. The contrasting increase of *napA* genes encoding a periplasmic nitrate reductase could be explained by the proliferation of bacteria performing aerobic denitrification. Aerobic denitrification has been shown repeatedly in other environments, with *napA* being the nitrate reductase used in this reaction (Ji *et al*. 2015). The nitrite reductase genes (*nirK* and *nirS*) did not show a clear overall dynamics pattern over time as the genes targeted by the different primers (nirS1, nirS2, nirS3 and nirK1, nirK2, nirK3) displayed diverse/contradictive changes with time.

The relative abundance of P cycling genes showed considerable temporal dynamics, and the genes involved in phytate degradation (*cphy*) and polyphosphate cycling (*ppk* and *ppx*) showed a decline with time. Phytate is the major P storage compound in plants and can represent a substantial proportion of seed dry weight (Lott *et al*. 2000). Phytate is degraded by plant phytases to release inorganic P (and other nutrients) for the developing seeds (Lott *et al*. 2000). However, our detection of bacterial *cphy* genes at 11 DAS suggests that bacteria are able to use the phytate stored in the seeds and might compete for released P during early germination. Our inability to detect the *cphy* phytase genes in the majority of the samples at 31 DAS (and 17 DAS; Fig. S9, Supporting Information) indicates that the seed-associated phytate is subsequently depleted. During germination, the hydrolysis of phytate releases inorganic orthophosphate that seemingly becomes available to bacteria as seen from the observed dynamics of the *ppk* and *ppx* genes involved in polyphosphate cycling. For comparison, a high occurrence of *ppx* and *ppk* genes was found in the maize rhizosphere, suggesting a possible enhancement of polyphosphate transformation and the availability of inorganic P in this environment (Li *et al*. 2014). According to the study by Li and co-workers, these genes were mainly distributed in Proteobacteria and Actinobacteria, taxa that dominate in the wheat seed-associated microbiota. Increases with time in the relative abundances of *phnK* involved in phosphonate utilization, the alkaline phosphatase genes *phoD* and *phoX*, as well as *pqq* involved in inorganic P solubilization might indicate a decrease in available P with time forcing the bacteria to exploit other organic P sources and increase solubilization of inorganic P. Genes involved in phosphonate utilization have previously been found in soil (Liu *et al*. 2018) and the *phnK* gene has even been found to be enriched in bacterial communities of the fungal hyphosphere, another nutrient cycling hotspot in the soil environment (Hao *et al*. 2020, Zhang *et al*. 2020). Along the same lines, high abundance of alkaline phosphatase genes, correlating with low available P (Olsen P), has been recorded in soil and rhizosphere environments (Acuña *et al*. 2016, Schneider *et al*. 2019), while the relative abundance of the *pqq g*ene is affected by several soil factors as pH (Zheng *et al*. 2019) and hence shows a less clear relation to P availability. Taken together, the dynamics of P cycling genes indicate an intense inorganic P and polyphosphate cycling by the seed-associated bacteria early after germination, driven by utilization of inorganic P released from the hydrolysis of phytate. Later, phytate depletion reduces the access to easily accessible P, forcing the seed-associated bacteria to use other organic P sources or solubilize inorganic P. Across all N and P cycling genes, the results show a larger effect of time than of the soil fertility level, supporting our first hypothesis.

### The microbial inoculants persisted throughout the experiment but showed very minor impact on the seed-associated bacterial communities

Microorganisms with plant beneficial traits are often added as inoculants to seeds to increase plant growth and health. Seed-coated microbial inoculants need to compete with indigenous communities in order to successfully colonize the seeds and benefit the plant. Therefore, we determined the persistence of the two inoculants, *P. bilaiae* and *B. simplex*, on the seeds in soils with different soil fertility levels. *Penicillium bilaiae*and*B. simplex* persisted on the seeds for the entire sampling period, supporting our second hypothesis. *Penicillium bilaiae* population size was neither affected by time nor by soil fertility level. Previous studies have demonstrated persistence of *P. bilaiae* for 3–4 weeks on wheat and maize seeds in laboratory pot or rhizobox experiments (Gómez-Muñoz *et al*. 2017, Hansen *et al*. 2020), and furthermore, *P. bilaiae* are found in as well as on wheat roots (Wakelin *et al*. 2004). Studies on inoculation with *B. simplex* on wheat are limited (Hassen and Labuschagne 2010, Hansen *et al*. 2020). In the present study, a transient increase in *B. simplex* was observed at 14 DAS. In contrast, Hansen *et al*. (2020) reported relatively stable populations of the current *B. simplex* strains on wheat seeds for up to 3 weeks. This discrepancy might be explained by a more frequent sampling in the present study allowing transient dynamics to be revealed. This further points to the importance of temporal dynamics when studying microbial interactions in natural systems. The inoculants, alone or in combination, did not affect the bacterial community alpha diversity, had a minor impact on the community compositions, and had insignificant effects on the occurrence of N and P cycling genes. Hence, there were no detectable unintended consequences of these inoculants in this natural seed-soil system, as the small changes in community structure were primarily due to the high amount of *B. simplex* introduced to the system. While the impact of inoculation with *P. bilaiae* and *B. simplex* is understudied, previous studies have found transient impact of seed-coated *B. subtilis* and *B. amyloliquifaciens* inoculants on tomato and lettuce rhizosphere microbiota (Erlacher *et al*. 2014, Qiao *et al*. 2017). These inoculated strains were biocontrol strains able to suppress soil-borne diseases and produce several antimicrobial metabolites such as polyketides and nonribosomal lipopeptides, causing a direct impact on the rhizosphere microbiota. The genomic backgrounds of the *B. simplex* strains, which were used here as part of a biofertilizer consortium, are not yet known, but information for another *B. simplex* strain 30N5 did not reveal genes for production of these antimicrobial compounds (Maymon *et al*. 2015). This may partly explain the observed small impact of *B. simplex* on the indigenous seed-associated microbiota. Moreover, this study was focusing on the persistence of the inoculants on the seed therefore not exploring their potential to colonize the newly formed roots and performing their deeds there. However, the persistence of *B. simplex* and *P. bilaiae* in the current study was assessed by direct DNA-targeted methods as differentiating the inoculants from indigenous soil species by plating was not possible. Development of methods to specifically determine the viability or metabolic activity of the inoculants under field conditions is highly needed to improve our understanding of the impact of inoculants on the seed, root and soil microbiota.

## Conclusion

The seed-associated communities were dominated by taxa previously recognized as seed-associated or endophytic, highlighting the importance of the seed for shaping its associated bacterial communities, even under field conditions. This notion is supported by a low impact of the soil fertility level on seed community composition in contrast to the considerable impact reported for the same fertilizer amendments on corresponding soil microbiota (van der Bom *et al*. 2018). A role of the seeds for the nutrition of their associated bacteria is indicated by the increased abundance over time of genes involved in organic N metabolism and ammonium oxidation, probably reflecting increased mineralization of plant-derived amino acids. Moreover, the high abundance of phytase genes after germination indicated bacterial mineralization of this seed storage compound. Indeed, it would be relevant to study whether bacterial phytase activity provides P for the plant or whether bacteria and plants compete for P. The inoculants had very limited impacts on the composition and potential functionality of the seed-associated bacterial community but have previously shown positive effects on wheat P uptake under laboratory conditions (Hansen *et al*. 2020). This highlights the potential of an indirect priming effect on plant performance by inoculants rather than a direct impact on microbial functionality, which should be addressed in future studies.

## Acknowledgments

The authors would like to thank Carsten Jørgensen, Knud-Erik S. Knudsen, Keld Skov Nielsen, Dusko Dimitrijevic, Birgitte Gjerde Bennedsen and Dorthe Thybo Ganzhorn for their assistance during the field trials; Frederik van der Bom for his help during the selection of fertilizer treatments and during sampling; Yongguan Zhu and Xinyuan Zhou for their contributions to the qPCR array analysis; and Lea Benedicte Skov Hansen for her support with the 16S rRNA amplicon sequence processing.

## Supplementary Material

fiac028_Supplemental_Files
