# Supplementary material for: Succession of the wheat seed-associated microbiome as affected by soil fertility level and introduction of Penicillium and Bacillus inoculants in the field
Source: FEMS Microbiol Ecol. 2022 Mar 14;98(3):fiac028. doi: 10.1093/femsec/fiac028 (PMC8951222; doi:10.1093/femsec/fiac028)
Supplement: fiac028_Supplemental_Files [file fiac028_supplemental_files.zip › FEMS_rev_Supplementary_Materials_seed_microbiome.docx]

**Supplementary Materials**

**Materials and methods**

**16S rRNA amplicon library preparation**

Amplicon libraries were prepared using two sequential PCR amplifications as described below. In a first step, the PCR mixes for amplifying the V3-V4 regions of the 16S rRNA gene were constituted by 4.0 µL of 5X Phusion© High Fidelity buffer (New England Biolabs, Inc., MA, USA), 0.2 µL Phusion© Hot Start II DNA Polymerase HF (2 mU, New England Biolabs, Inc., MA, USA), 0.4 µL of PCR Nucleotide Mix (10 mM/dNTP, Roche, Mannheim, Germany) 1 µL of each primer (10 µM), 3 µL of the DNA extract as template and water to a total volume of 20 µL. The PCR reactions were performed according to the following conditions: an initial activation of the hot-start polymerase at 98°C for 2 min, followed by 25 cycles of denaturation at 98°C for 30 s, annealing at 52°C for 30 s and extension at 72°C for 30 s, with a final extension at 72°C for 5 min. Amplified PCR products were quantified using a Qubit 3.0 fluorometer (Invitrogen, Life Technologies, Nærum, Denmark) with a Qubit® dsDNA HS Assay Kit (range 0.2-100 ng; Invitrogen, Life Technologies, Nærum, Denmark) and purified using the AmPure XP Bead Purification Kit (Beckman Coulter, Copenhagen, Denmark) according to the manufacturer’s instructions and applying 16 µL of Agencourt AMPure XP per reaction. In a second step, indexes (8 bp) were added to DNA fragments using PCR mixes as in the first step but with the following exceptions: 1.0 µL of each barcoded primer (10 µM) and 1 µL of PCR product from the first PCR. Second PCR reactions were performed in the following way: initial activation of the hotstart polymerase at 98°C for 2 min, followed by 10 cycles of denaturation at 98°C for 30 s, annealing at 55°C for 30 s and extension at 72°C for 30 s, with final extension at 72°C for 5 min. All amplifications were performed in a Peltier Thermal Cycler (DNA Engine DYAD™, USA). The obtained fragments were approximately 587 bp long including primers and indexes. A second purification was performed using the AmPure XP Bead Purification Kit (Beckman Coulter, Copenhagen, Denmark) as previously described. The concentration of the purified second PCR amplicons was measured by a Qubit 3.0 fluorometer (Invitrogen, Life Technologies, Nærum, Denmark) using a Qubit® dsDNA HS Assay Kit (range 0.2-100 ng; Invitrogen, Life Technologies, Nærum, Denmark) and equal amounts of DNA were pooled to create the final amplicon library. Fifty µL of the pooled library were further cleaned and concentrated to 30 µL using the AmPure XP Bead Purification Kit (Beckman Coulter, Copenhagen, Denmark) according to the manufacturer’s instructions (40 µL of Agencourt AMPure XP; 200 µL of 80 % ethanol for washing steps).

**Table S2** Primer sequences for genes involved in nitrogen and phosphorus cycling

| Gene name | Encoded protein | Predicted length (bp) | Forward Primer | Forward sequence (5'-3') | Reverse Primer | Reverse Sequence (5'-3') | Reference |
| --- | --- | --- | --- | --- | --- | --- | --- |
| *amoA*1 | ammonia monooxygenase α subunit (Archaea) | 635 | Arch-amoAF | STAATGGTCTGGCTTAGACG | Arch-amoAR | GCGGCCATCCATCTGTATGT | (Francis et al. 2005) |
| *amoA*2 | ammonia monooxygenase α subunit (Bacteria) | 490 | amoA-1F | GGGGTTTCTACTGGTGGT | amoA-2R | CCCCTCKGSAAAGCCTTCTT | (Rotthauwe et al. 1997) |
| *amo*B | ammonia monooxygenase β subunit | 501 | amoBMf | TGGTAYGACATKAWATGG | amoBMr | RCGSGGCARGAACATSGG | (Calvó and Garcia-Gil 2004) |
| *bpp* | β-propeller phytase | 160-200 | BPP-F | GACGCAGCCGAYGAYCCNGCNITNTGG | BPP-R | CAGGSCGCANRTCIACRTTRTT | (Huang et al. 2009) |
| *cphy* | ruminal cysteine phytase | 380-400 | Cphy-F | GTGGACCTRCGRMARGARWCICA | Cphy-R | GTCCGACCATTGCCTGCYTCRCARTGRAMRTGIADCCA | (Huang et al. 2011) |
| *gcd* | quinoprotein glucose dehydrogenase | 300 | Gcd-F | ATCGCGTTCGGGCCGGACG | Gcd-R | ATSAGRTTSAGCTCGTCCCA | (Zheng et al. 2018) |
| *gdh*A | glutamate dehydrogenase | 240 | GdhA-F | GCCATCGGYCCWTACAAGGG | GdhA-R | ATGTCRCCNGCCGGAACGTC | (Zheng et al. 2018) |
| *hao/hzo* | hydroxylamine oxidoreductase | 218 | hao/hzo_cl2aF1 | TGTCACATGGGTGTAGACCA | hao/hzo_cl2aR1 | ACCTGGAACATACCCAT | (Nunoura et al. 2013) |
| *hzo* | hydrazine oxidase | 224 | HzoQPCR1F | AAGACNTGYCAYTGGGGWAAA | HzoQPCR1R | GACATACCCATACTKGTRTANACNGT | (Long et al. 2013) |
| *hzs*A | hydrazine synthase α subunit | 260 | hzsA_1597F | WTYGGKTATCARTATGTAG | hzsA_1857R | AAABGGYGAATCATARTGGC | (Shen et al. 2013) |
| *hzs*B | hydrazine synthase β subunit | 381 | HSBeta296F | ARGGHTGGGGHAGYTGGAAG | HSBeta742R | GTYCCHACRTCATGVGTCTG | (Wang et al. 2012) |
| *nap*A | periplasmic nitrate reductase | 490 | napAf1 | CTGGACIATGGGYTTIAACCA | napAr1 | CCTTCYTTYTCIACCCACAT | (Feng et al. 2011) |
| *nar*G | nitrate reductase α chain | 110 | 1960m2F | TAYGTSGGGCAGGARAAACTG | 2050m2R | CGTAGAAGAAGCTGGTGCTGT | (López-Gutiérrez et al. 2004) |
| *nas*A | assimilatory nitrate reductase catalytic subunit | 750-800 | nas964 | CARCCNAAYGCNATGGG | nasA1735 | ATNGTRTGCCAYTGRTC | (Allen et al. 2001) |
| *nif*H | nitrogenase iron protein | 400 | nifHF | AAAGGYGGWATCGGYAARTCCACCAC | nifHRb | TGSGCYTTGTCYTCRCGGATBGGCAT | (Rösch and Bothe 2005) |
| *nir*K1 | nitrite reductase (NO-forming) | 514 | nirK1F | GGMATGGTKCCSTGGCA | nirK5R | GCCTCGATCAGRTTRTGGTT | (Braker et al. 1998) |
| *nir*K2 | nitrite reductase (NO-forming) | 450 | nirKC1F | ATGGCGCCATCATGGTNYTNCC | nirKC1R | TCGAAGGCCTCGATNARRTTRTG | (Wei et al. 2015) |
| *nir*K3 | nitrite reductase (NO-forming) | 400 | nirKC2F | TGCACATCGCCAACGGNATGTWYGG | nirKC2R | GGCGCGGAAGATGSHRTGRTCNAC | (Wei et al. 2015) |
| *nir*S1 | nitrite reductase (NO-forming) | 425 | nirScd3AF | GTSAACGTSAAGGARACSGG | nirSR3cd | GASTTCGGRTGSGTCTTGA | (Jung et al. 2011) |
| *nir*S2 | nitrite reductase (NO-forming) | 400 | nirSC1F | ATCGTCAACGTCAARGARACVGG | nirSC1R | TTCGGGTGCGTCTTSABGAASAG | (Wei et al. 2015) |
| *nir*S3 | nitrite reductase (NO-forming) | 400 | nirSC2F | TGGAGAACGCCGGNCARGTNTGG | nirSC2R | GATGATGTCCACGGCNACRTANGG | (Wei et al. 2015) |
| *nos*Z1 | nitrous-oxide reductase | 267 | nosZ2F | CGCRACGGCAASAAGGTSMSSGT | nosZ2R | CAKRTGCAKSGCRTGGCAGAA | (Henry et al. 2006) |
| *nos*Z2 | nitrous-oxide reductase | 454 | nosZ-F | CGYTGTTCMTCGACAGCCAG | nosZ1622R | CGSACCTTSTTGCCSTYGCG | (Throbäck et al. 2004) |
| *nxr*A | nitrite oxidoreductase α subunit | 322 | F1370-F1 | CAGACCGACGTGTGCGAAAG | F2843-R2 | TCCACAAGGAACGGAAGGTC | (Wertz et al. 2008) |
| *phn*K | phosphonate transport system ATP-binding protein | 366 | PhnK-F | CATCGTCGGCGAATCCGG | PhnK-R | TGCTGCATGCCGCCGGAAAA | (Zheng et al. 2018) |
| *pho*D | alkaline phosphatase D | 370 | ALPS-F730 | CAGTGGGACGACCACGAGGT | ALPS-R1101 | GAGGCCGATCGGCATGTCG | (Sakurai et al. 2008) |
| *pho*X | alkaline phosphatase/Pho regulon | 600 | phoX2-F | GARGAGAACWTCCACGGYTA | phoX2-R | GATCTCGATGATRTGRCCRAAG | (Sebastian and Ammerman 2009) |
| *ppk* | polyphosphate kinase | 296 | Ppk-F | GACCCGAABGTRCTBGCSAT | Ppk-R | TTATAATTNCCSGTNCCNA | (Zheng et al. 2018) |
| *ppx* | Exopolyphosphatase | 310 | Ppx-F | TGCATCTGGCGGACGGCCT | Ppx-R | AGATCCGCCGCCAATATCA | (Zheng et al. 2018) |
| *pqq*C | pyrroloquinoline-quinone synthase | 300 | PqqC-F | AACCGCTTCTACTACCAG | PqqC-R | GCGAACAGCTCGGTCAG | (Zhang et al. 2017) |
| *ure*C | Urease | 340 | ureC-F | AAGMTSCACGAGGACTGGGG | ureC-R | AGRTGGTGGCASACCATSAGCAT | (Koper et al. 2004) |
| 16S | ribosomal RNA gene sequence (reference gene) | 393 | F515 | GTGCCAGCMGCCGCGG | R907 | CCGTCAATTCMTTTRAGTTT | (Zhou et al. 2011) |

**Table S3** PERMANOVA results for 16S rRNA amplicon data obtained from seed-associated samples (11 DAS to 31 DAS) using soil fertility level, time, inoculum and the different possible interactions between those three as factors. The analysis was based on Bray-Curtis dissimilarity index and 1,000 permutations.

| Factor | Df | SumOfSqs | R^2^ | F | P |
| --- | --- | --- | --- | --- | --- |
| Fertility Level | 2 | 1.598 | 0.03554 | 4.6613 | 0.001 |
| Time | 3 | 12.140 | 0.27000 | 23.6096 | 0.001 |
| Inoculum | 3 | 1.129 | 0.02510 | 2.1949 | 0.002 |
| Fertility Level:Time | 6 | 2.930 | 0.06516 | 2.8488 | 0.001 |
| Fertility Level:Inoculum | 6 | 1.096 | 0.02438 | 1.0660 | 0.283 |
| Time:Inoculum | 9 | 1.700 | 0.03781 | 1.1022 | 0.251 |
| Fertility Level:Time:Inoculum | 18 | 2.945 | 0.06550 | 0.9546 | 0.658 |
| Residual | 125 | 21.425 | 0.47651 |  |  |
| Total | 172 | 44.963 | 1.00000 |  |  |

**Table S4** PERMANOVA results for nitrogen and phosphorus cycling genes using soil fertility level, time, inoculum and the different possible interactions between those three as factors. The analysis was based on Bray-Curtis dissimilarity index and 1,000 permutations.

| **Factor** | **Df** | **SumOfSqs** | **R^2^** | **F** | **P** |
| --- | --- | --- | --- | --- | --- |
| Time | 3 | 7.103 | 0.39770 | 49.3086 | 0.001 |
| Fertility Level | 2 | 0.394 | 0.02204 | 4.0992 | 0.004 |
| Inocolum | 3 | 0.269 | 0.01507 | 1.8678 | 0.067 |
| Time:Fertility Level | 6 | 1.004 | 0.05624 | 3.4863 | 0.001 |
| Time:Inoculum | 9 | 0.476 | 0.02665 | 1.1014 | 0.349 |
| Fertility Level:Inoculum | 6 | 0.672 | 0.03764 | 2.3334 | 0.004 |
| Time:Fertility Level:Inoculum | 18 | 1.075 | 0.06021 | 1.2441 | 0.145 |
| **Residual** | 143 | 6.866 | 0.38446 |  |  |
| **Total** | 190 | 17.86 | 1.00000 |  |  |


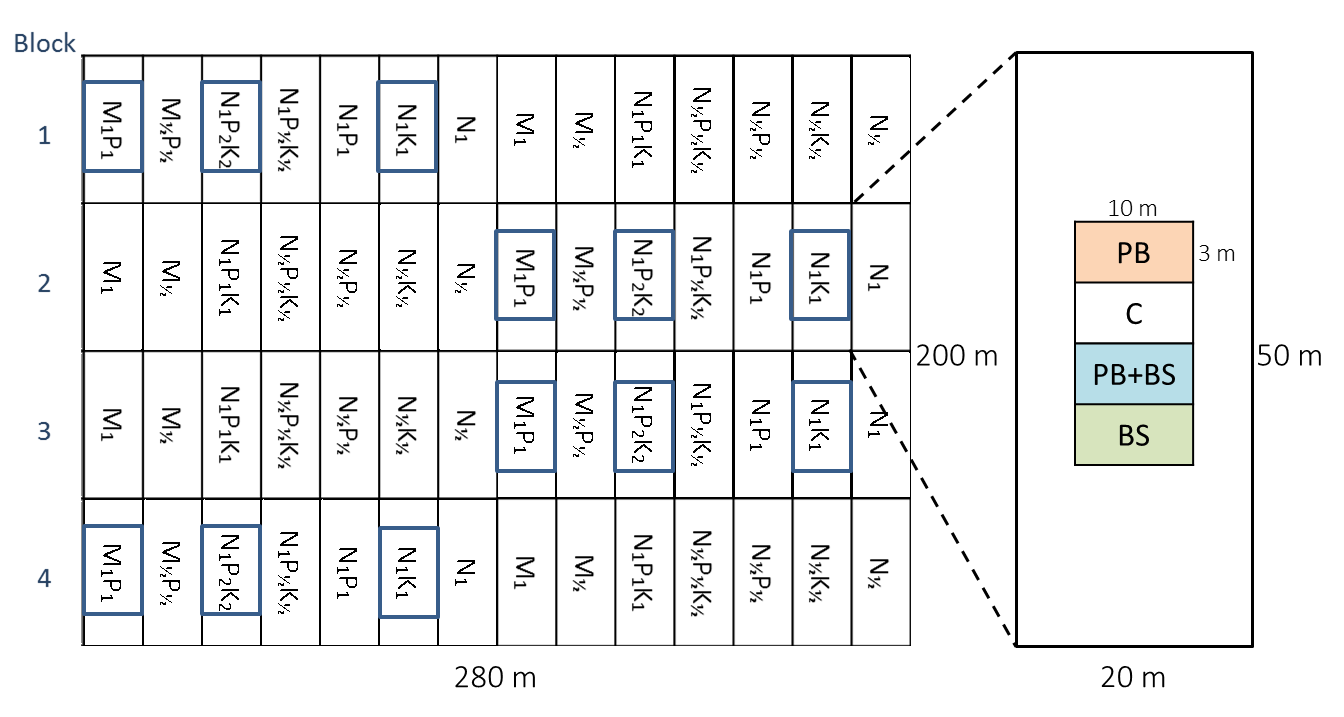


**Fig. S1** Schematic presentation of the field experiment. The three soil fertility levels (N_1_K_1_, N_1_P_2_K_2_ and M_1_P_1_) with four replications are indicated with blue frames (on the left). The mini-plots were randomized within each soil fertility level plot (to the right).


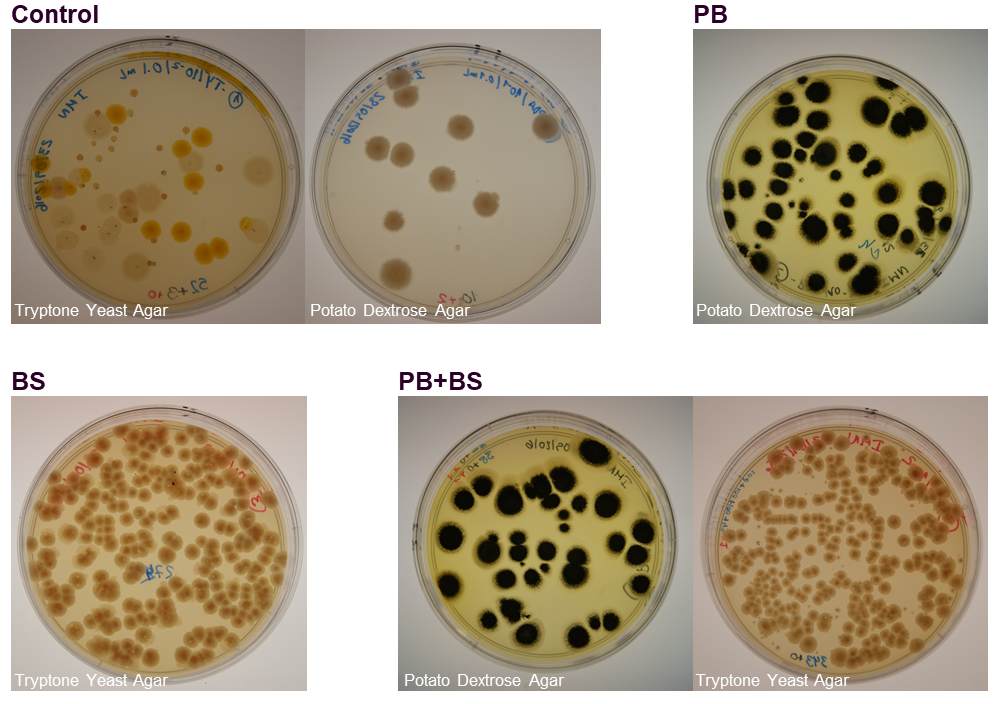


**Fig. S2** Pictures of the plates resulting from the seed organism recovery after treatment with the different inocula. Control – Pictures of the bacterial (in Tryptone Yeast agar) and fungal (in Potato Dextrose Agar) natural community on seed; PB – Picture of the fungal community recovered from the treatment with *P. bilaiae* depicting only *P. bilaiae* colonies; BS - Picture of the bacterial community recovered from the treatment with *B. simplex* depicting only *B. simplex* colonies; PB+BS – Pictures of the fungal (in Potato Dextrose Agar) and the bacterial (in Tryptone Yeast agar) communities retrieved from the combined treatment with *P. bilaiae* and *B. simplex* depicting only *P. bilaiae* and *B. simplex* colonies, respectively, in each type of media.


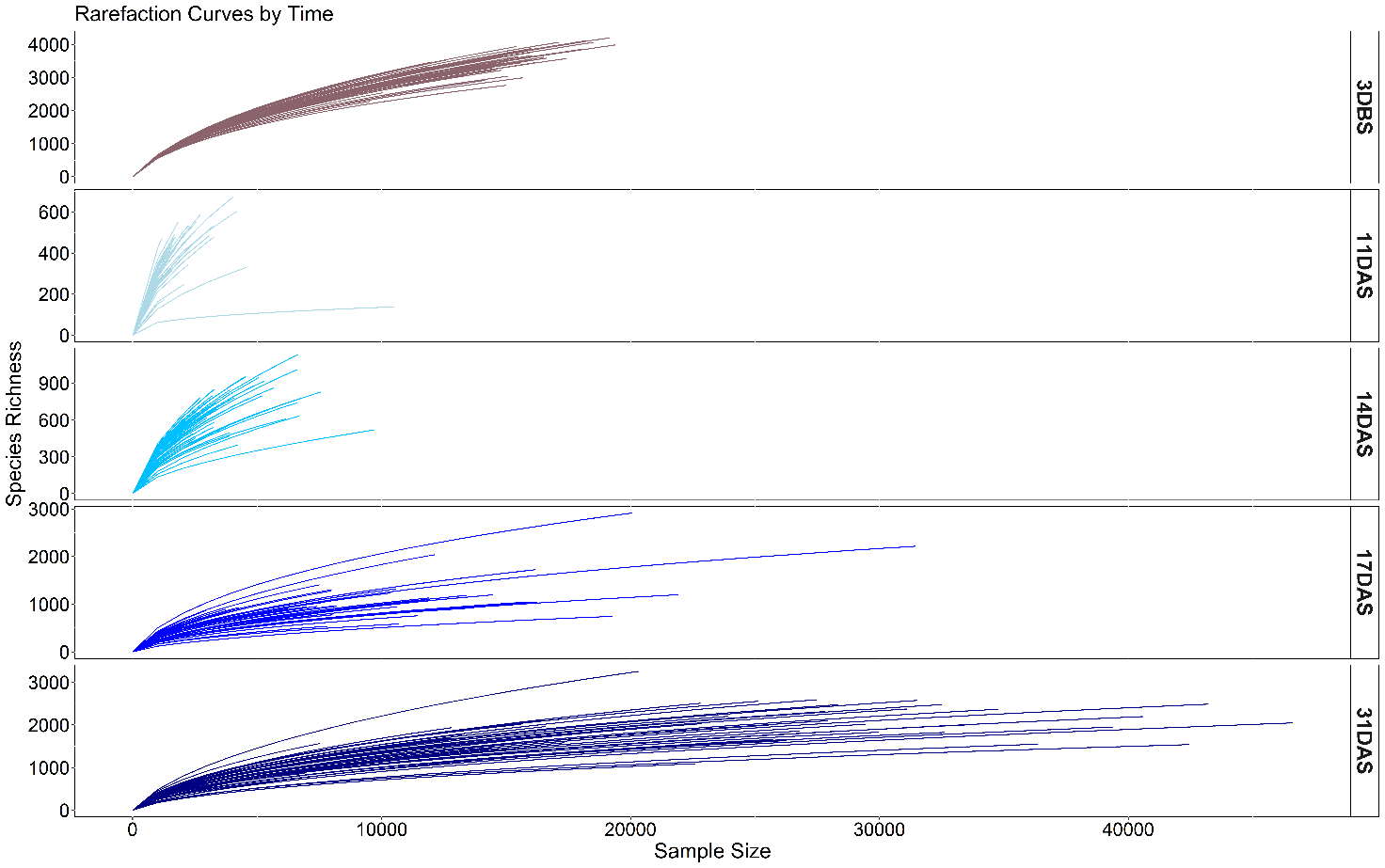


**Fig. S3** Rarefaction curves computed at zOTU level for each individual sample. Panels depict rarefaction curves for samples collected in at each time point. DBS: days before sowing; DAS: days after sowing.

10

10

12

12

**Fig. S4** Daily precipitation and average daily temperature from the day of sowing (23/09/2016) until the last sampling date (24/10/2016). Arrows represent the four sampling dates (11, 14, 17 and 31 days after sowing) and the numbers above represent the growth stage of winter wheat according to the Zadock scale (stage 10: first leave through the coleoptile; stage 12: two-leaves stage).

**Fig. S5** Shoot (A) and root (B) dry matter biomass harvested 17 day after sowing. Values presented here are means with standard error bars (n=4). Lower letters indicate statistical difference between soil fertility level.

**
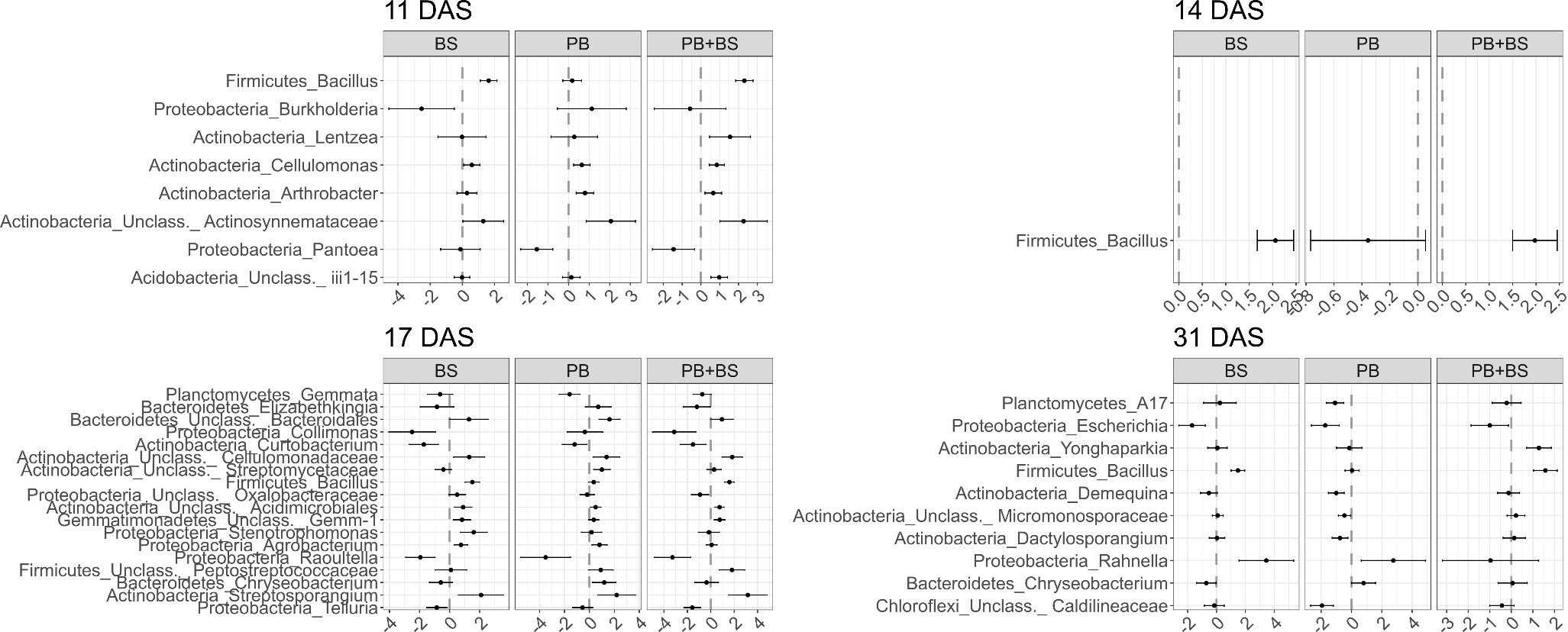
**

**Fig. S6** Genera showing significant changes in relative abundance expressed as differential abundance between inoculant treatment and control at the four sampling times. The changes in relative abundance are estimated using Corncob, while controlling for fertility level. p_adj_ < 0.05. Positive values indicate increase of a genus in seeds that have been inoculated. DAS: days after sowing.


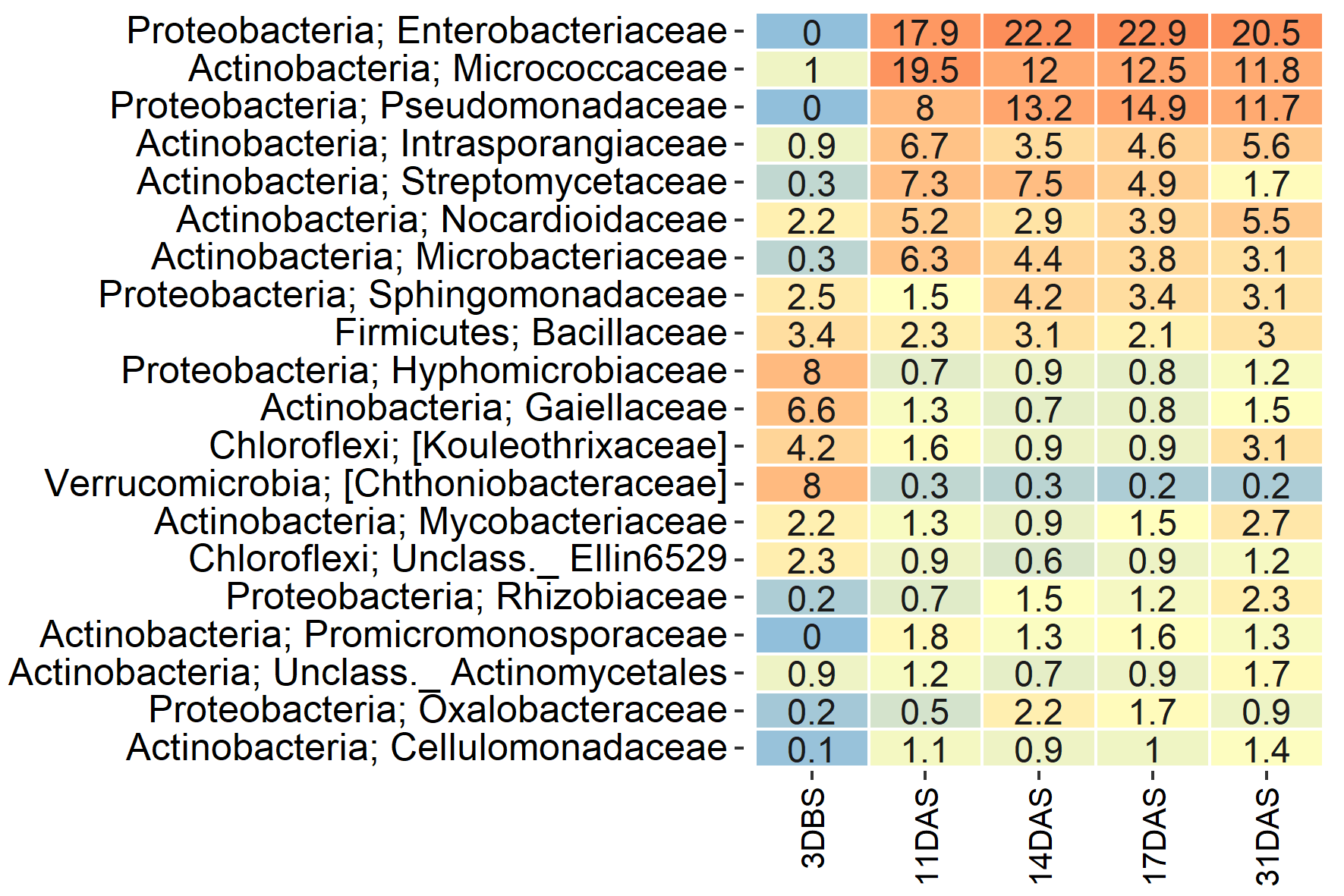


**Fig S7.** Heat map showing the relative abundance (%) of the 20 most abundant seed-associated bacterial families at 3 days before sowing (DBS), and 11, 14, 17 and 31 days after sowing (DAS). The data are mean values of determinations for the different soil fertility levels and inoculations. The relative abundances of ASVs are indicated by a color gradient going from blue (low abundance) to red (high abundance).

**
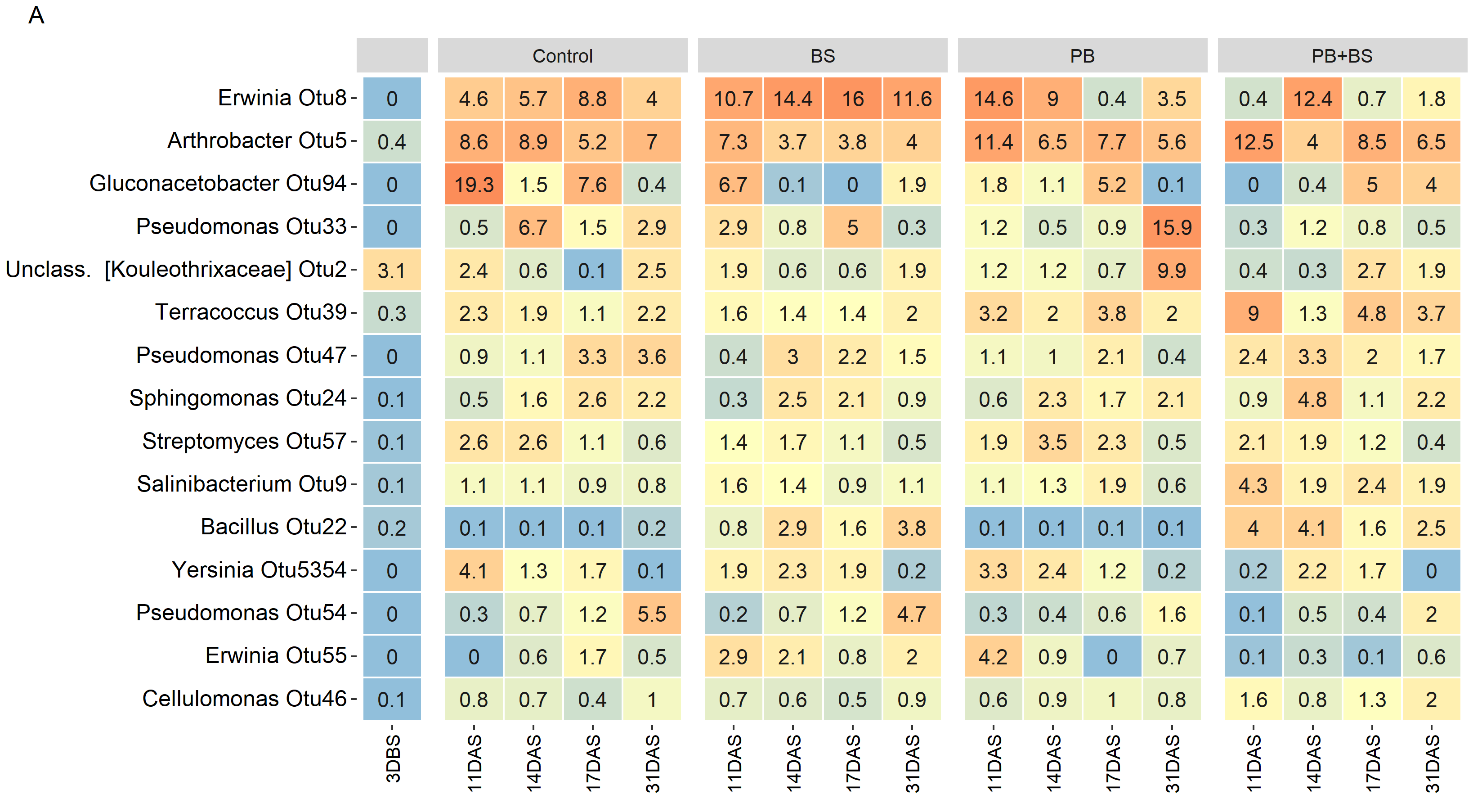
**

**
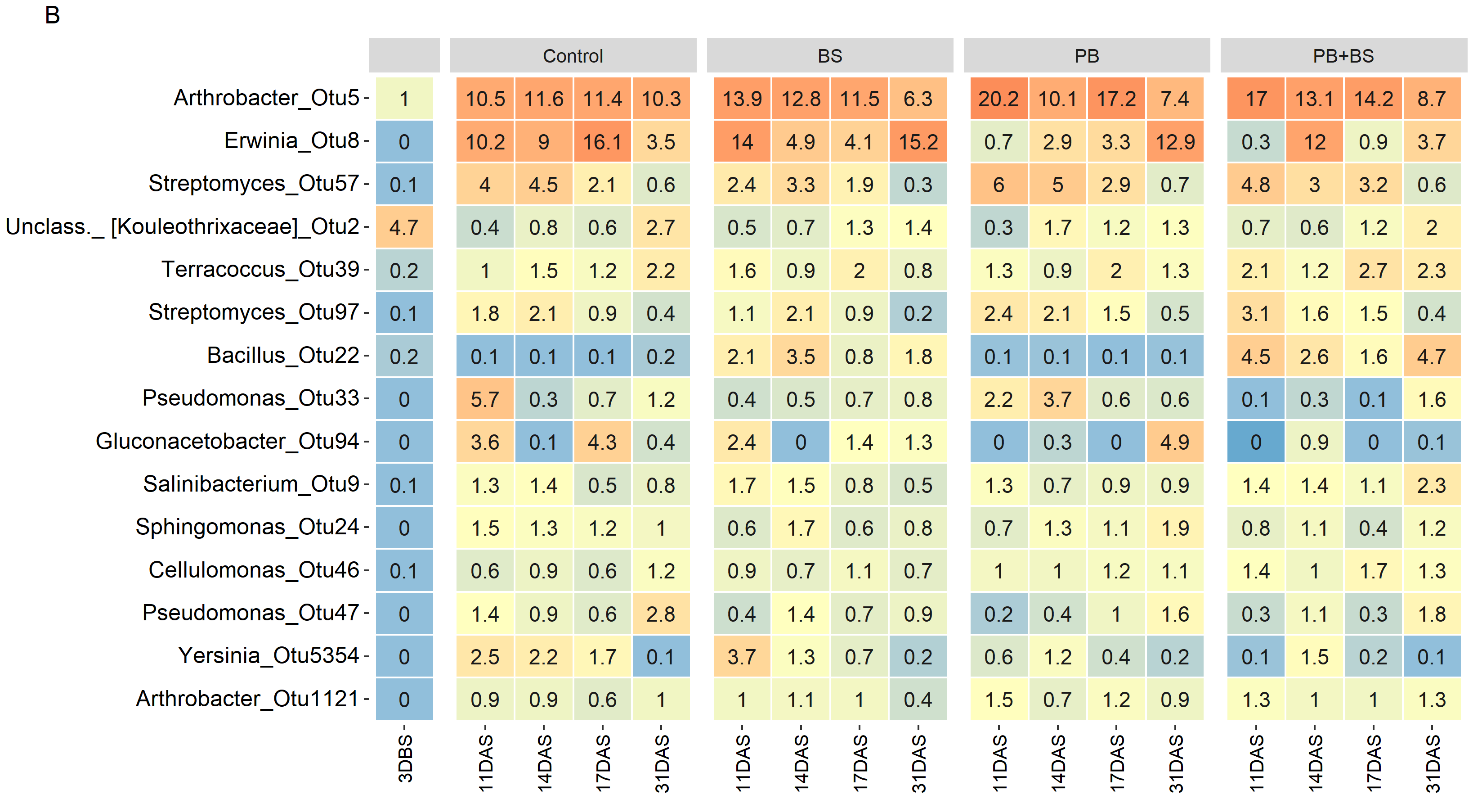
**

**Fig S8.** Relative abundances of the 15 most abundant zOTUs in bulk soil (3DBS) and seed-associated communities in the different soil fertility levels, A) N1P2K2 and B) M1P1. Relative abundances are mean values sampling times. 3 days before sowing (DBS) bulk soil samples (n = 48), and 11, 14, 17 and 31 days after sowing (DAS) (n = 4). The zOTUs: Otu8, Otu9, Otu22, Otu24, Otu33, Otu47 and Otu55 were detected on original seed samples. *Bacillus* OTU22 were only detected in seeds inoculated in *B. simplex.*


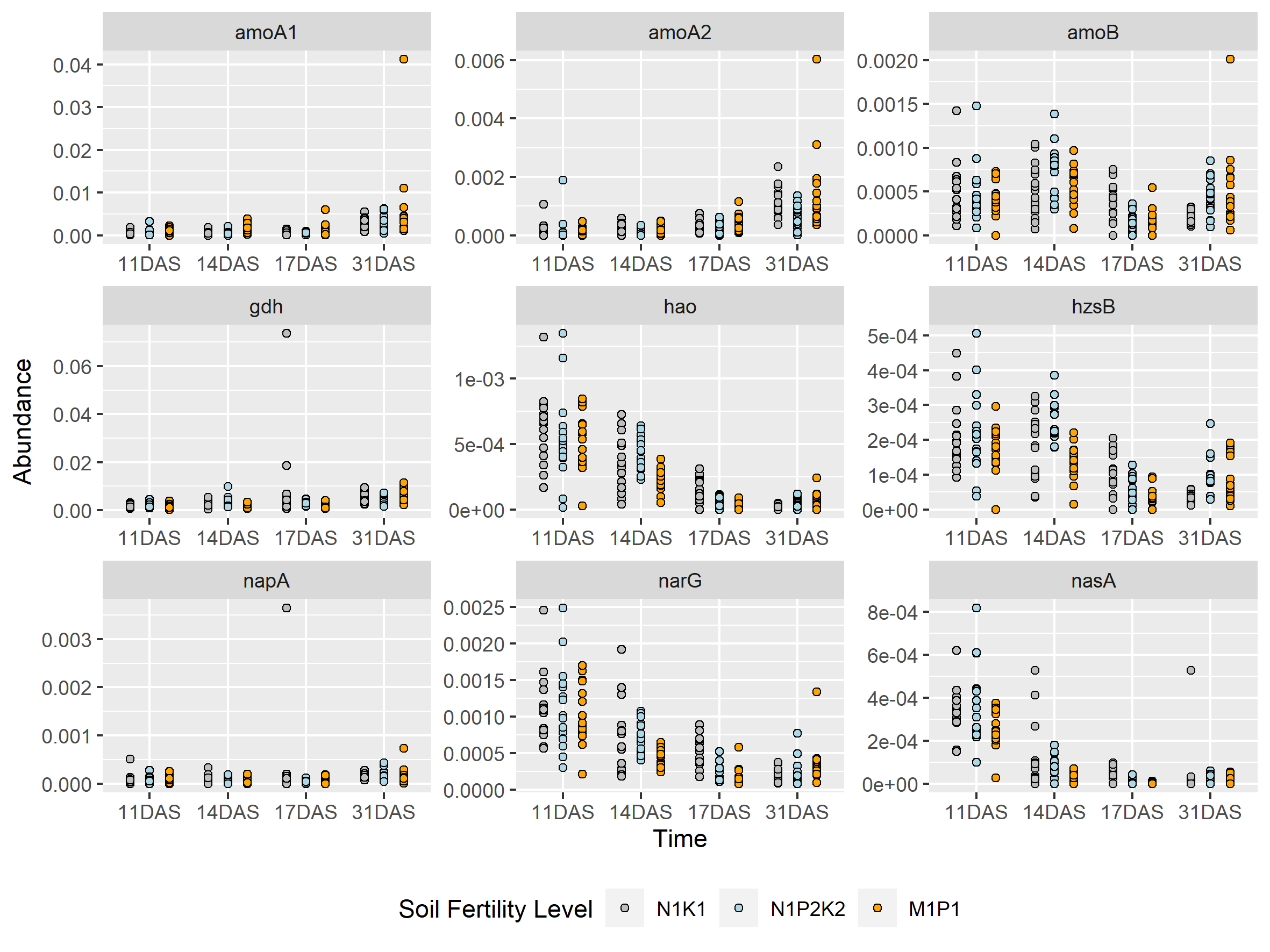


**Fig S9.** Relative abundances of genes across sampling times. Gene abundances were normalized to the 16S rRNA abundance. Colours represent the soil fertility levels (treatments). Each dot represents a sample.


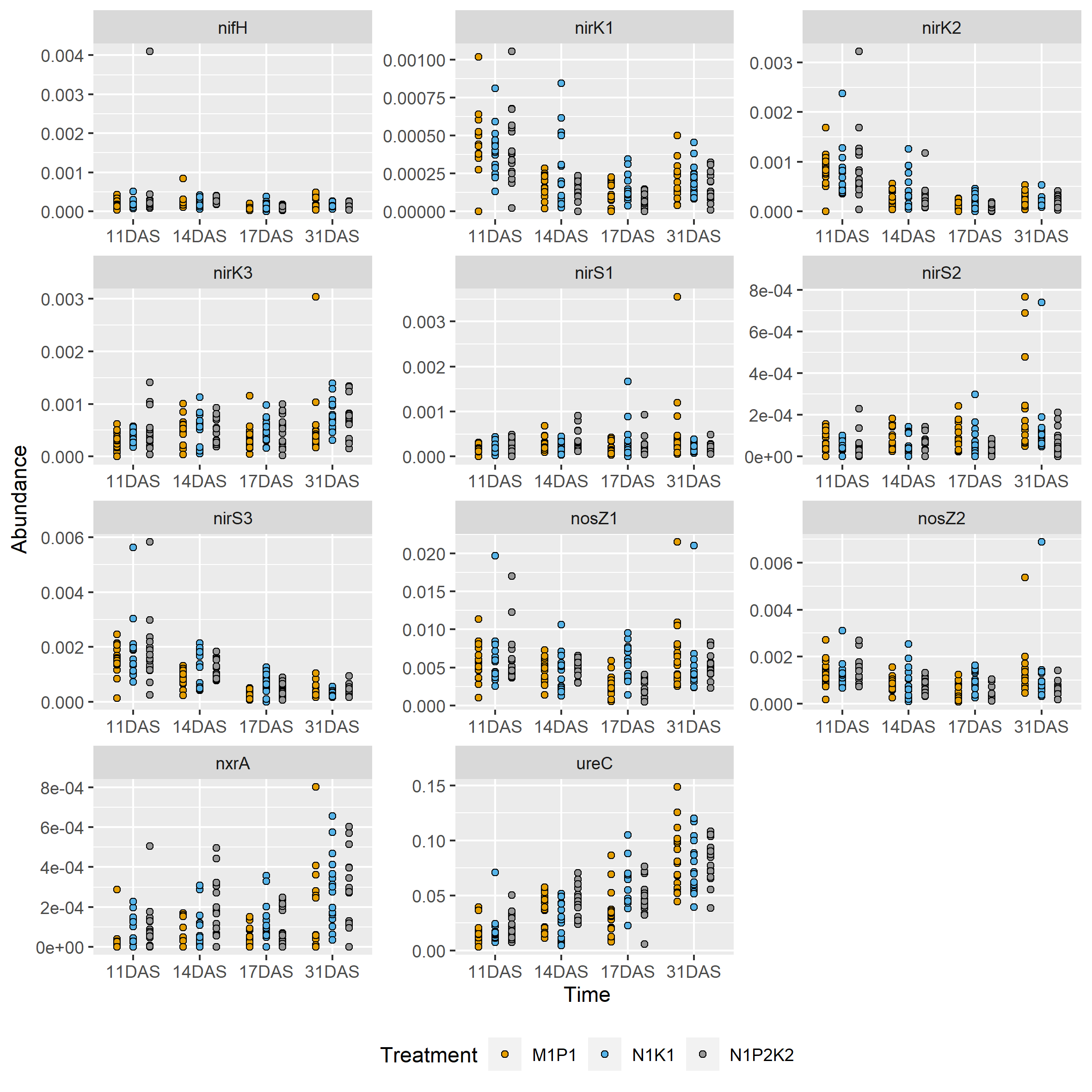


**Fig S9 continued.**


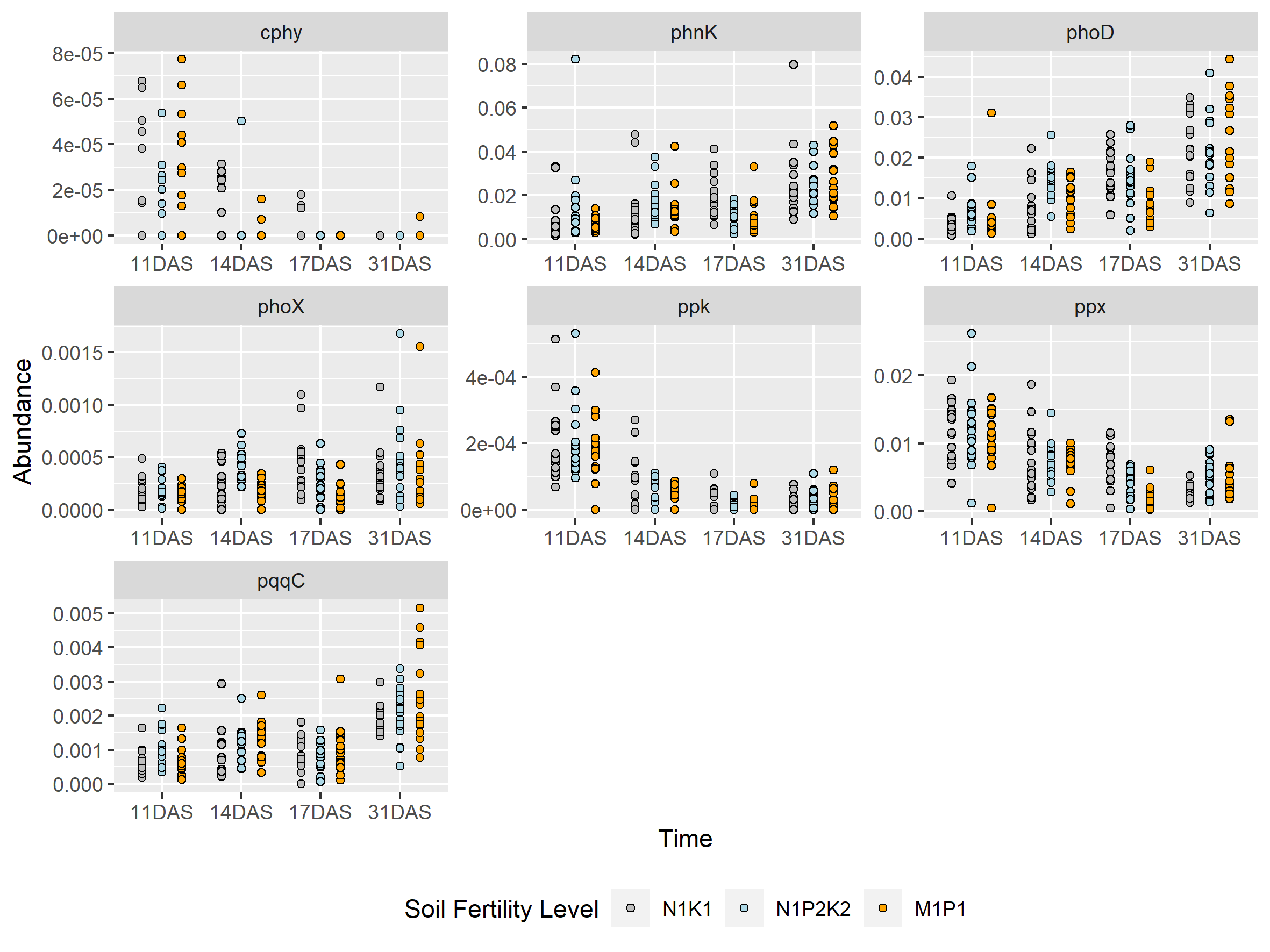


**Fig S9 continued.**

**References**

Allen AE, Booth MG, Frischer ME, et al (2001) Diversity and detection of nitrate assimilation genes in marine bacteria. Appl Environ Microbiol 67:5343–5348. https://doi.org/10.1128/aem.67.11.5343-5348.2001

Braker G, Fesefeldt A, Witzel KP (1998) Development of PCR primer systems for amplification of nitrite reductase genes (nirK and nirS) to detect denitrifying bacteria in environmental samples. Appl Environ Microbiol 64:3769–3775. https://doi.org/10.1128/aem.64.10.3769-3775.1998

Calvó L, Garcia-Gil LJ (2004) Use of amoB as a new molecular marker for ammonia-oxidizing bacteria. J Microbiol Methods 57:69–78. https://doi.org/10.1016/j.mimet.2003.11.019

Feng WW, Liu JF, Gu JD, Mu BZ (2011) Nitrate-reducing community in production water of three oil reservoirs and their responses to different carbon sources revealed by nitrate-reductase encoding gene (napA). Int Biodeterior Biodegrad 65:1081–1086. https://doi.org/10.1016/j.ibiod.2011.05.009

Francis CA, Roberts KJ, Beman JM, et al (2005) Ubiquity and diversity of ammonia-oxidizing archaea in water columns and sediments of the ocean. Proc Natl Acad Sci U S A 102:14683–14688. https://doi.org/10.1073/pnas.0506625102

Henry S, Bru D, Stres B, et al (2006) Quantitative detection of the nosZ gene, encoding nitrous oxide reductase, and comparison of the abundances of 16S rRNA, narG, nirK, and nosZ genes in soils. Appl Environ Microbiol 72:5181–5189. https://doi.org/10.1128/AEM.00231-06

Huang H, Shi P, Wang Y, et al (2009) Diversity of beta-propeller phytase genes in the intestinal contents of grass carp provides insight into the release of major phosphorus from phytate in nature. Appl Environ Microbiol 75:1508–1516. https://doi.org/10.1128/AEM.02188-08

Huang H, Zhang R, Fu D, et al (2011) Diversity, abundance and characterization of ruminal cysteine phytases suggest their important role in phytate degradation. Environ Microbiol 13:747–757. https://doi.org/10.1111/j.1462-2920.2010.02379.x

Jung J, Yeom J, Kim J, et al (2011) Change in gene abundance in the nitrogen biogeochemical cycle with temperature and nitrogen addition in Antarctic soils. Res Microbiol 162:1018–1026. https://doi.org/10.1016/j.resmic.2011.07.007

Koper TE, El-Sheikh AF, Norton JM, Klotz MG (2004) Urease-encoding genes in ammonia-oxidizing bacteria. Appl Environ Microbiol 70:2342–2348. https://doi.org/10.1128/AEM.70.4.2342-2348.2004

Long A, Heitman J, Tobias C, et al (2013) Co-occurring anammox, denitrification, and codenitrification in agricultural soils. Appl Environ Microbiol 79:168–176. https://doi.org/10.1128/AEM.02520-12

López-Gutiérrez JC, Henry S, Hallet S, et al (2004) Quantification of a novel group of nitrate-reducing bacteria in the environment by real-time PCR. J Microbiol Methods 57:399–407. https://doi.org/10.1016/j.mimet.2004.02.009

Nunoura T, Nishizawa M, Kikuchi T, et al (2013) Molecular biological and isotopic biogeochemical prognoses of the nitrification-driven dynamic microbial nitrogen cycle in hadopelagic sediments. Environ Microbiol 15:3087–3107. https://doi.org/10.1111/1462-2920.12152

Rösch C, Bothe H (2005) Improved assessment of denitrifying, N2-fixing, and total-community bacteria by terminal restriction fragment length polymorphism analysis using multiple restriction enzymes. Appl Environ Microbiol 71:2026–2035. https://doi.org/10.1128/AEM.71.4.2026-2035.2005

Rotthauwe JH, Witzel KP, Liesack W (1997) The ammonia monooxygenase structural gene amoa as a functional marker: Molecular fine-scale analysis of natural ammonia-oxidizing populations. Appl Environ Microbiol 63:4704–4712. https://doi.org/10.1128/aem.63.12.4704-4712.1997

Sakurai M, Wasaki J, Tomizawa Y, et al (2008) Analysis of bacterial communities on alkaline phosphatase genes in soil supplied with organic matter. Soil Sci Plant Nutr 54:62–71. https://doi.org/10.1111/j.1747-0765.2007.00210.x

Sebastian M, Ammerman JW (2009) The alkaline phosphatase PhoX is more widely distributed in marine bacteria than the classical PhoA. ISME J 3:563–572. https://doi.org/10.1038/ismej.2009.10

Shen L dong, Liu S, Lou L ping, et al (2013) Broad distribution of diverse anaerobic ammonium-oxidizing bacteria in Chinese agricultural soils. Appl Environ Microbiol 79:6167–6172. https://doi.org/10.1128/AEM.00884-13

Throbäck IN, Enwall K, Jarvis Å, Hallin S (2004) Reassessing PCR primers targeting nirS, nirK and nosZ genes for community surveys of denitrifying bacteria with DGGE. FEMS Microbiol Ecol 49:401–417. https://doi.org/10.1016/j.femsec.2004.04.011

Wang Y, Zhu G, Harhangi HR, et al (2012) Co-occurrence and distribution of nitrite-dependent anaerobic ammonium and methane-oxidizing bacteria in a paddy soil. FEMS Microbiol Lett 336:79–88. https://doi.org/10.1111/j.1574-6968.2012.02654.x

Wei W, Isobe K, Nishizawa T, et al (2015) Higher diversity and abundance of denitrifying microorganisms in environments than considered previously. ISME J 9:1954–1965. https://doi.org/10.1038/ismej.2015.9

Wertz S, Poly F, Le Roux X, Degrange V (2008) Development and application of a PCR-denaturing gradient gel electrophoresis tool to study the diversity of Nitrobacter-like nxrA sequences in soil. FEMS Microbiol Ecol 63:261–271. https://doi.org/10.1111/j.1574-6941.2007.00416.x

Zhang B, Penton CR, Xue C, et al (2017) Soil depth and crop determinants of bacterial communities under ten biofuel cropping systems. Soil Biol Biochem 112:140–152. https://doi.org/10.1016/J.SOILBIO.2017.04.019

Zheng B, Zhu Y, Sardans J, et al (2018) QMEC: a tool for high-throughput quantitative assessment of microbial functional potential in C, N, P, and S biogeochemical cycling. Sci China Life Sci 61:1451–1462. https://doi.org/10.1007/s11427-018-9364-7

Zhou J, Wu L, Deng Y, et al (2011) Reproducibility and quantitation of amplicon sequencing-based detection. ISME J 5:1303–1313. https://doi.org/10.1038/ismej.2011.11
